# B Cells and IL-21-Producing Follicular Helper T Cells Cooperate to Determine the Dynamic Alterations of Premetastatic Tumor Draining Lymph Nodes of Breast Cancer

**DOI:** 10.34133/research.0346

**Published:** 2024-03-29

**Authors:** Xinrui Mao, Xinyu Tang, Hong Pan, Muxin Yu, Sihan Ji, Wen Qiu, Nan Che, Kai Zhang, Zhendong Huang, Yunshan Jiang, Ji Wang, Zhaoyun Zhong, Jiaming Wang, Mingduo Liu, Mingkang Chen, Wenbin Zhou, Shui Wang

**Affiliations:** ^1^ Department of Breast Surgery, The First Affiliated Hospital with Nanjing Medical University, 300 Guangzhou Road, Nanjing 210029, China.; ^2^ Jiangsu Key Lab of Cancer Biomarkers, Prevention and Treatment, Jiangsu Collaborative Innovation Center For Cancer Personalized Medicine, School of Public Health, Nanjing Medical University, Nanjing 211166, China.; ^3^ Department of Immunology, Nanjing Medical University, Nanjing 211166, China.; ^4^ Department of Rheumatology and Immunology, The First Affiliated Hospital with Nanjing Medical University, 300 Guangzhou Road, Nanjing 210029, China.; ^5^ Pancreatic Center & Department of General Surgery, The First Affiliated Hospital with Nanjing Medical University, Nanjing 210029, Jiangsu, China.; ^6^ Pancreas Institute of Nanjing Medical University, Nanjing 210029, Jiangsu, China.; ^7^ Department of Pathology, The First Affiliated Hospital with Nanjing Medical University, 300 Guangzhou Road, Nanjing 210029, China.; ^8^ Department of Ophthalmology, The First Affiliated Hospital with Nanjing Medical University, 300 Guangzhou Road, Nanjing 210029, China.

## Abstract

Metastasis is the major cause of cancer-related death, and lymph node is the most common site of metastasis in breast cancer. However, the alterations that happen in tumor-draining lymph nodes (TDLNs) to form a premetastatic microenvironment are largely unknown. Here, we first report the dynamic changes in size and immune status of TDLNs before metastasis in breast cancer. With the progression of tumor, the TDLN is first enlarged and immune-activated at early stage that contains specific antitumor immunity against metastasis. The TDLN is then contracted and immunosuppressed at late stage before finally getting metastasized. Mechanistically, B and follicular helper T (Tfh) cells parallelly expand and contract to determine the size of TDLN. The activation status and specific antitumor immunity of CD8^+^ T cells in the TDLN are determined by interleukin-21 (IL-21) produced by Tfh cells, thus showing parallel changes. The turn from activated enlargement to suppressed contraction is due to the spontaneous contraction of germinal centers mediated by follicular regulatory T cells. On the basis of the B-Tfh-IL-21-CD8^+^ T cell axis, we prove that targeting the axis could activate TDLNs to resist metastasis. Together, our findings identify the dynamic alterations and regulatory mechanisms of premetastatic TDLNs of breast cancer and provide new strategies to inhibit lymph node metastasis.

## Introduction

Cancer metastasis is the major cause of cancer-related death and seizes the attention of cancer researches as a largely unknown complicated process [1,2]. Usually, surgeons depend on physical and imaging examinations to remove tumor-draining lymph nodes (TDLNs) when there are palpable enlarged lymph nodes. However, enlarged lymph nodes are not necessarily pathologically positive, and a substantial portion of them prove to be reactive lymph nodes [3,4]. Therefore, it is important to understand the biology of TDLNs and the changes they may experience during the progression of tumors.

Premetastatic microenvironment has been a prevailing concept in the past few decades, as emerging data have provided evidence that under the influence of the primary tumor, there has established an immunosuppressed, tumor-tolerant microenvironment in distant organs before metastatic seeding [5,6]. The existence of premetastatic microenvironment has been reported in multiple organs including the lungs, liver, brain, and bones [7–9]. Conditioned by soluble factors, extracellular vesicles or migratory immune cells from the tumor microenvironment, immunoinhibitory compartments including regulatory T cells (T_regs_), and myeloid-derived suppressor cells accumulate in distant organs to prepare them for metastatic seeding [10–12]. In the setting of lymph node, previous researches have also described a microenvironment similar to those in other organs, with impaired T cell and dendritic cell functions accompanied by accumulation of T_regs_ [13–24]. Others have focused on the emergence of tumor-promoting or tumor-attracting factors like lymphangiogenesis, secretion of chemokines, and collagen accumulation [25–28]. These findings are consistent with the common sense that a suppressed immune status may favor tumor survival and naturally relate premetastatic lymph nodes to an immunosuppressed microenvironment.

However, as peripheral lymphoid tissues, lymph nodes also perform their biological role of generating adaptive immunity in response to local stimuli including tumors [29,30]. When antigens are carried to the draining lymph node by lymphoid fluid or antigen-presenting cells, specific immune responses arise in these lymph nodes and further spread to become systemic immunity [31]. It has been established that TDLNs are crucial for the local and systemic antitumor immune responses induced by chemotherapy, radiotherapy, and immunotherapy [32–34]. In preclinical studies, lymph nodes seem to possess intrinsic resistance toward tumor cells [35–38]. Descriptive clinical studies have also reported increases in dendritic cells, CD8^+^ T cells, and B cells in negative TDLNs in some tumor types, but the significance of these changes remains unclear [39–41]. All these preclinical and clinical evidence seem sectional and fragmented, adding much complexity to the premetastatic microenvironment of TDLNs especially in the poorly investigated breast cancer.

Therefore, despite the multiple significance of lymph nodes in tumor staging, prognosis, and immunogenesis, the premetastatic change of TDLNs still remains controversial and largely unknown. Here, in this study, we first report the dynamic changes in size and immune status of TDLNs before metastasis in breast cancer, which is different from the previously reported immunosuppressed microenvironment. With the progression of the primary breast tumor, the TDLNs were first enlarged and immune-activated at early stage and then contracted and immunosuppressed at late stage before finally metastasized. Mechanistically, the dynamic alterations in size and antitumor immunity were regulated by the B-follicular helper T (Tfh) cell-interleukin-21 (IL-21)-CD8^+^ T cell axis. Importantly, by targeting the axis, the premetastatic TDLNs could be reactivated to resist metastasis. Our findings made a new and more comprehensive description of the premetastatic microenvironment in TDLNs and provided clues for future treatment strategies to eliminate lymph node metastasis with their intrinsic immunity.

## Results

### TDLNs of breast cancer undergo enlargement and subsequent contraction before metastasis

**Fig. 1.**
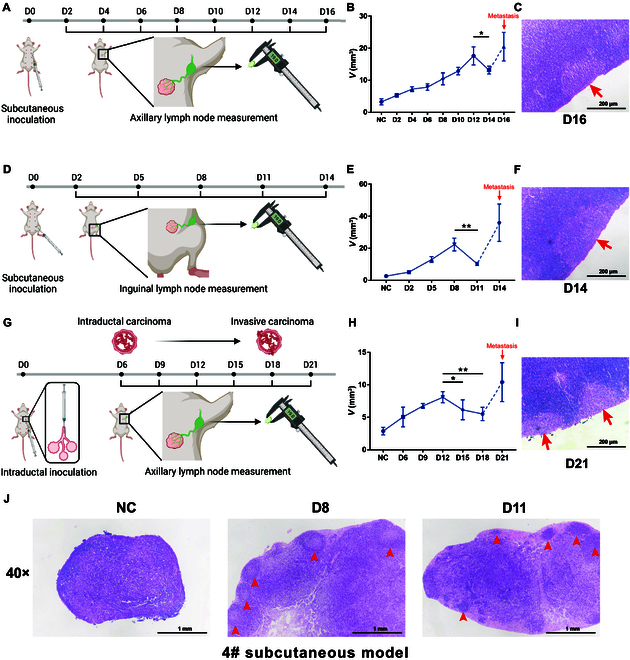
TDLNs of breast cancer undergo enlargement and subsequent contraction before metastasis. (A to C) In the 2# subcutaneous model, 4T1 cells were injected subcutaneously into the left second mammary fat pad of mice. The TDLN, axillary lymph node, was obtained at different time points for measurement and pathological examination. Axillary lymph nodes of normal mice were obtained as NC. Experimental design (A), change in volume of TDLN (B), and H&E staining of metastatic TDLN (C) are shown (*n* = 4 mice per time point). (D to F and J) In the 4# subcutaneous model, 4T1 cells were injected subcutaneously into the left fourth mammary fat pad of mice. The TDLN, inguinal lymph node, was obtained at different time points for measurement and pathological examination. Inguinal lymph nodes of normal mice were obtained as NC. Experimental design (D), change in volume of TDLN (E), H&E staining of metastatic TDLN (F), and premetastatic TDLNs (arrowheads indicate germinal centers) (J) are shown (*n* = 4 mice per time point). (G to I) In the 2# intraductal model, 4T1 cells were injected into the mammary duct of the left second mammary gland of mice. The TDLN, axillary lymph node, was obtained at different time points for measurement and pathological examination. Experimental design (G), change in volume of TDLN (H), and H&E staining of metastatic TDLN (I) are shown (*n* = 5 mice per time point). All experiments were repeated at least twice independently. Data are means ± SD. Unpaired Student’s *t* test was used to evaluate statistical significance. *P < 0.05; **P < 0.01.

In an effort to understand in detail how the TDLN changes with the progression of breast cancer, it was first observed how the size of TDLN changed in murine subcutaneous tumor models. After inoculation of 4T1 breast cancer cells into the second (2#)/fourth (4#) pair of mammary fat pad, respectively, their TDLNs (axillary/inguinal lymph node) were obtained at different time points after inoculation (Fig. 1A and D). Pathological examination was carried out to determine the times of lymph node metastasis occurrence in the 2 models [day 16 (D16) in 2# subcutaneous model and D14 in 4# subcutaneous model] (Fig. 1C and F). Interestingly, before the time of metastasis, the 2 models showed a similar drift in the size of their TDLNs. After a gradual enlargement phase of about 10 d, the lymph node underwent a transient but significant contraction before subsequent metastatic enlargement (Fig. 1B and E and Fig. S1A and B).

Although we have observed the same drift in size of TDLN in 2 independent subcutaneous tumor models, tumor inoculation into mammary fat pads can only mimic the progression of invasive carcinoma. To better simulate human breast cancer progression from intraductal carcinoma to invasive carcinoma, we further adopted an intraductal tumor model to replicate the experiment [42]. By microinjecting 4T1 cells into the mammary duct, an early-stage breast cancer model was established that developed from intraductal carcinoma into invasive carcinoma (Fig. 1G and Fig. S2). Again, the time of metastasis was determined as D21 (Fig. 1I). Equally, the same trend of TDLN size was observed in this model as in subcutaneous models. The drift from gradual enlargement to transient contraction before metastasis was slower and more moderate, consistent with the slow progression of the primary tumor in this model (Fig. 1H and Fig. S1C). By hematoxylin and eosin (H&E) sections, the change of TDLN in the 4# subcutaneous model were further displayed (Fig. 1J). The enlargement and contraction of the TDLN were accompanied by obvious structural remodeling. Taken together, these results verified by multiple models that the TDLN of breast cancer underwent an enlargement–contraction drift before metastasis. As the 4# subcutaneous model showed the most stable and distinct change in size and the inguinal lymph node is larger so as to provide more cells for analysis, the 4# subcutaneous model was adopted for further investigation.

### The alteration in antitumor ability of TDLN is parallel to its dynamic alteration in size

**Fig. 2.**
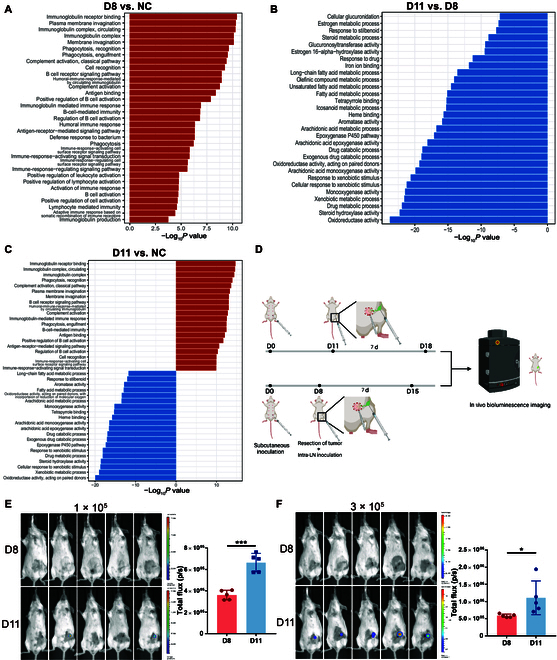
The antitumor ability of TDLN is parallel to its dynamic alteration in size. (A to C) 4T1 cells were injected subcutaneously into the left fourth mammary fat pad of mice. TDLNs were obtained on D8 and D11 for RNA-seq. Inguinal lymph nodes of normal mice were obtained as NC. GO pathways enriched in differentially expressed genes of mice’s TDLNs are shown between D8 and NC (A), D11 and D8 (B), and D11 and NC (C), respectively (*n* = 2 mice per group). The *y* axis shows the top 30 enriched categories, with up-regulated pathways represented in red and down-regulated pathways represented in blue. (D to F) 4T1 cells were injected subcutaneously into the left fourth mammary fat pad of mice. On D8 or D11, the primary tumor was resected, and different amounts of 4T1-luc cells were injected into the subcapsular sinus of TDLN to simulate lymph node (LN) metastasis. Seven days after tumor resection and intra-lymph-node injection, mice were subjected to bioluminescent assay, and the inguinal flank area was selected to analyze total bioluminescence flux (D). The bioluminescent images and the total flux in the inguinal area are shown, with 1 × 10^5^ (E) or 3 × 10^5^ (F) 4T1-luc cells injected into the TDLN, respectively (*n* = 5 mice per group). All experiments were repeated at least twice independently. Data are means ± SD. Unpaired Student’s *t* test was used to evaluate statistical significance. *P < 0.05; ***P < 0.001.

Since a recent clinical study has associated larger TDLN size with stronger immune responses in the lymph node [4], we next sought to determine whether the alteration of TDLN size corresponded to the alteration of immune status. Because, in the 4# subcutaneous model, D8 was the peak of TDLN enlargement, while D11 showed the steep premetastatic contraction, the TDLNs of these 2 contrary time points were subjected to RNA sequencing (RNA-seq). Gene Set Enrichment Analysis of D8 versus NC (normal control) revealed that all basic cell processes were up-regulated in TDLNs on D8 compared to normal lymph nodes, with many immune-related processes among the top enriched processes (Fig. 2A and Fig. S3A). Given that lymph nodes are nearly exclusively consisted of immune cells, it could be assumed that the D8 TDLN was generally immune-activated since the immune cells up-regulated their basic cell processes. When D11 was compared with D8, however, all of the top enriched gene sets on D11 showed a descending trend, indicating a relatively immunosuppressed status on D11 (Fig. 2B and Fig. S3B). When comparing D11 with NC, all immune-related processes were still up-regulated although there were some down-regulated pathways, supporting the notion that the D11 shrunk TDLN is only relatively less activated than D8 but still more active than the NC baseline (Fig. 2C and Fig. S3C).

To confirm the primitive presumption from RNA-seq, we further tested the antitumor capacity of TDLNs in vivo. Using an intra-lymph-node injection technique [18], the lymph nodes were directly inoculated with tumor cells and tested for their antitumor immunity (Fig. 2D). After excision of the primary tumor and inoculation of 4T1-luc cells into the TDLN to simulate translocation of tumor cells into the lymph node, only the D11 TDLN was permissive to tumor cell colonization, while there was no detectable tumor colonization in the D8 TDLN (Fig. 2E and Fig. S4). Even when the injected tumor burden was raised to 300,000 cells and the D11 TDLN was totally invaded, the D8 TDLN was still able to eliminate the incoming tumor cells (Fig. 2F and Fig. S4). The in vivo assay indicated that the D8 enlarged TDLN was indeed immune-activated and possessed strong antitumor immunity. Only when the TDLN turned to be contracted on D11, the premetastatic microenvironment suitable for metastatic colonization had formed in the TDLN, which was followed by metastasis occurrence shortly afterward on D14. Taken together, the results above suggested a correlation between the antitumor immune status and the size of a TDLN.

### The changing antitumor ability of TDLN is mediated by tumor-specific CD8^+^ T cells

**Fig. 3.**
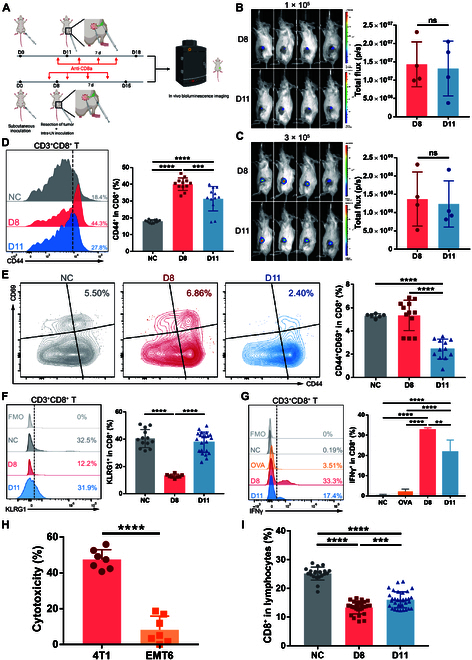
The changing antitumor ability of TDLN is mediated by tumor-specific CD8^+^ T cells. (A to C) 4T1 cells were injected subcutaneously into the left fourth mammary fat pad of mice. On D8 or D11, the primary tumor was resected, and different amounts of 4T1-luc cells were injected into the subcapsular sinus of TDLN to simulate lymph node metastasis. Starting from 1 day before tumor resection and intra-lymph-node injection (D7 or D10), each mouse was injected intraperitoneally 4 times with anti-CD8a antibody every 2 d. Seven days after tumor resection and intra-lymph-node injection, mice were subjected to bioluminescent assay and the inguinal flank area was selected to analyze total bioluminescence flux (A). The bioluminescent images and the total flux in the inguinal area are shown, with 1 × 10^5^ (B) or 3 × 10^5^ (C) 4T1-luc cells injected into the TDLN, respectively (*n* = 4 mice per group). (D to F and I) 4T1 cells were injected subcutaneously into the left fourth mammary fat pad of mice. TDLNs were obtained on D8 and D11 for flow cytometry (*n* = 12 mice per group). Inguinal lymph nodes of normal mice were obtained as NC. (G) Mice were subcutaneously inoculated with 4T1 cells and lymph nodes were obtained as in (D) to (F) (*n* = 3 to 4 mice per group). Lymph node cells were stimulated with 4T1 tumor antigen peptide AH1 or irrelevant antigen peptide OVA in vitro for 12 h and then harvested for flow cytometry. The percentages of IFNγ^+^ cells in CD8^+^ T cells are shown. (H) Mice were subcutaneously inoculated with 4T1 cells and lymph nodes were obtained as in (D) to (F) (*n* = 7 mice per group). Lymph node cells were cocultured with 4T1 or EMT6 tumor cells for 24 h, and cytotoxicity was assessed by LDH release assay. All experiments were repeated at least twice independently. Data are means ± SD. Unpaired Student’s *t* tests (B, C, and H) and one-way ANOVA tests followed by Tukey’s post hoc test (D to G and I) were used to evaluate statistical significance. **P < 0.01; ***P < 0.001; ****P < 0.0001. ns, not significant.

CD8^+^ T and natural killer (NK) cells are the 2 major types of immune cells responsible for antitumor immunity. As lymph nodes mainly consist of lymphocytes and contain few innate immune cells like NK cells, we postulated that CD8^+^ T cell might be the direct mediator of the changing antitumor ability here in premetastatic TDLNs. After in vivo ablation of CD8^+^ T cells, the intra-lymph-node inoculation experiment was repeated (Fig. 3A). This time, both D8 and D11 lymph nodes showed evident metastatic invasion with no significant difference between the 2 groups (Fig. 3B and C). This confirmed that the changing antitumor ability was directly mediated by CD8^+^ T cells and possibly reflected the change in antitumor capacity of CD8^+^ T cells.

Therefore, we focused on CD8^+^ T cells to determine their alteration in activation status during the premetastatic period. The level of CD44, which is an important activation marker, was first determined in CD8^+^ T cells on D8 and D11 (Fig. S5A). In line with the previous RNA-seq and in vivo antitumor assay results, the expression of CD44 on CD8^+^ T cells was much higher on D8 than NC, which decreased significantly on D11 (Fig. 3D and Fig. S5B). Among the CD44^+^ activated CD8^+^ T cells, we further examined the expression of early activation marker CD69 (Fig. S5A). The proportion of CD44^+^CD69^+^ early-activated cells was significantly reduced on D11, confirming that there was active production of newly activated CD8^+^ T cells on D8 but not on D11 (Fig. 3E and Fig. S5C). On the other hand, when the terminal differentiation marker killer cell lectin-like receptor G1 (KLRG1) was examined on CD8^+^ T cells, a much higher proportion of KLRG1^+^ cells were observed on D11 than D8. This means that CD8^+^ T cells have entered a terminal-differentiated, potentially exhausted state on D11 (Fig. 3F). Together with the inactive production of CD69^+^ early-activated cells, this explains the low overall activation level and poor antitumor capacity of CD8^+^ T cells on D11. Thus, by the examination of different activation markers, we validated that the activation level of CD8^+^ T cells changed in parallel to the size and antitumor ability of TDLN, further verifying the presumption that CD8^+^ T cells mediated the antitumor immunity of TDLN.

Last, to better relate the activation level of CD8^+^ T cells to the antitumor immunity of TDLN, we tested whether this activation was tumor-specific. Using the 4T1-tumor-specific antigen peptide AH1 [43,44], we determined the proportion of tumor-specific CD8^+^ T cells by in vitro stimulation. After coculture with the cells from D8 TDLN, the tumor peptide AH1 was able to stimulate a considerable amount of interferon-γ^+^ (IFNγ^+^) tumor-specific CD8^+^ T cells, while the irrelevant control antigen peptide ovalbumin (OVA) showed no effect of stimulation (Fig. 3G). Moreover, by specific cytotoxicity assay, we confirmed that the activated CD8^+^ T cells from D8 possessed killing capacity only toward 4T1 cells rather than other tumor cells (Fig. 3H). Altogether, these results showed that the activation status of CD8^+^ T cells changed throughout the premetastatic phase of TDLN to mediate the changing specific antitumor immunity.

### Expansion and contraction of B cells determine the size of TDLN and regulate CD8^+^ T cell activation

Interestingly, despite the elevated activation level of CD8^+^ T cells, the D8 TDLN conversely contained a much lower percentage of CD8^+^ T cells than NC, and the trend sustained until D11 (Fig. 3I). This could not account for the enlargement of TDLN on D8 either. The contradictory facts prompted us to speculate that rather than directly activated by the tumor, CD8^+^ T cells were passively activated by other immune compartments that underwent direct activation and massive expansion, thus reducing the relative percentage of CD8^+^ T cells in TDLN.

**Fig. 4.**
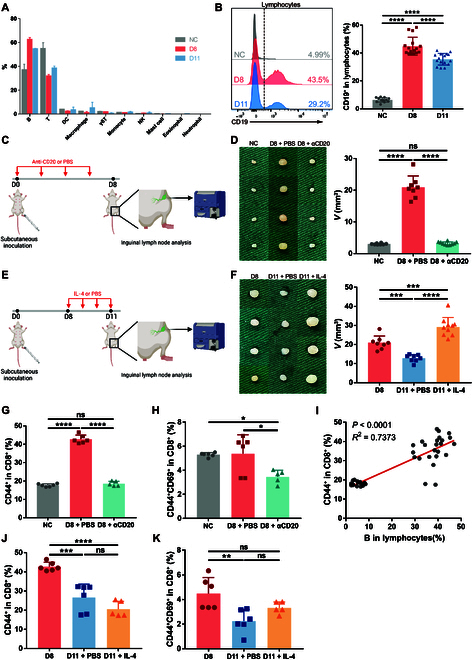
Expansion and contraction of B cells determine the size of TDLN and regulate CD8^+^ T cell activation. (A) Percentages of immune cells in TDLNs estimated by CIBERSORTx with the RNA-seq data above. DC, dendritic cells. (B) 4T1 cells were injected subcutaneously into the left fourth mammary fat pad of mice. TDLNs were obtained on D8 and D11 for flow cytometry (*n* = 12 mice per group). Inguinal lymph nodes of normal mice were obtained as NC. (C, D, G, and H) 4T1 cells were injected subcutaneously into the left fourth mammary fat pad of mice. Starting from D0, anti-CD20 antibody or PBS was injected subcutaneously 4 times at the inguinal area of each mouse every 2 d (D0, D2, D4, and D6) (C). TDLNs were obtained on D8 for measurement (D) (*n* = 8 to 10 mice per group) and flow cytometry (G and H) (*n* = 5 to 6 mice per group). (E, F, J, and K) 4T1 cells were injected subcutaneously into the left fourth mammary fat pad of mice. Starting from D8, recombinant IL-4 or PBS was injected subcutaneously 4 times at the inguinal area of each mouse daily (D8, D9, D10, and D11) (E). TDLNs were obtained on D11 for measurement (F) (*n* = 8 to 10 mice per group) and flow cytometry (J and K) (*n* = 5 to 6 mice per group). (I) Linear regression and correlation between B cells and CD44^+^CD8^+^ T cells were done with paired data from Fig. 3D, (B), (G), and Fig. S6. All experiments were repeated at least twice independently. Data are means ± SD. One-way ANOVA tests followed by Tukey’s post hoc test and *t* test for Pearson *r* were used to evaluate statistical significance. *P < 0.05; **P < 0.01; ***P < 0.001; ****P < 0.0001.

To determine the immune compartment, we carried out CIBERSORTx with the TDLN RNA-seq data to display the percentages of immune cells in TDLNs at different time points (Fig. 4A). Of the 2 main types of lymphocytes that compose lymph nodes, B cells were significantly augmented on D8 in comparison to NC, while T cells correspondingly decreased. In accordance with the expansion of B cells, many up-regulated immune-related processes in Gene Set Enrichment Analysis directed to the activation of B cells (Fig. 2A). On D11, B cells decreased to a relatively lower proportion than D8 but still above the NC baseline, which exactly coincide with the change in size of TDLN (Fig. 4A). The alteration of B cells was validated by flow cytometry (Fig. 4B). The trend was further verified in the previous H&E staining results, with evident emergence and swelling of germinal centers on D8 and contraction of germinal centers on D11 (Fig. 1J, arrowheads indicate germinal centers).

Given that breast cancer is known to markedly activate B cells especially in lymph nodes [45–48], we suppose that B cells were first activated and expanded in TDLN to cause the enlargement and contraction of the lymph node. To confirm the supposition, we in vivo ablated B cells with αCD20 antibody on D8 (Fig. 4C) and stimulated B cells with IL-4 on D11 (Fig. 4E), respectively. Successful ablation and stimulation were verified by flow cytometry (Fig. S6). Ablation of B cells on D8 led to a steep shrink of the TDLN to a size comparable to NC, which means that there is no other cell cluster directly activated and expanded by the tumor (Fig. 4D). On the other hand, stimulation of B cells on D11 enlarged the TDLN to a size even larger than D8 (Fig. 4F). Thus, B cells proved to be the determinant of TDLN size in the premetastatic changes.

We next sought to determine whether B cells are correlated to the activation status of CD8^+^ T cells. In the D8 TDLN ablated of B cells, both CD44^+^ activated CD8^+^ T cells (Fig. 4G) and CD44^+^CD69^+^ early-activated CD8^+^ T cells (Fig. 4H) reduced significantly among CD8^+^ T cells. Correlation analysis revealed a significant positive correlation between CD44^+^CD8^+^ T and B cells (Fig. 4I). These results suggest that B cells not only actively expand and contract to determine the TDLN size but also correlate with CD8^+^ T cell activation status to regulate the antitumor immunity of TDLN.

### Tfh cells expand and contract along with B cells to directly regulate CD8^+^ T cells

Although ablation of B cells on D8 abrogated CD8^+^ T activation, it was strange that stimulation of B cells with IL-4 on D11 failed to cause a higher activation level of CD8^+^ T cells (Fig. 4J and K). This reminded us that B cells might not be the direct regulator and determinant of CD8^+^ T cell activation, and there could be other B-cell-related compartments directly regulating the activation status of CD8^+^ T cells.

**Fig. 5.**
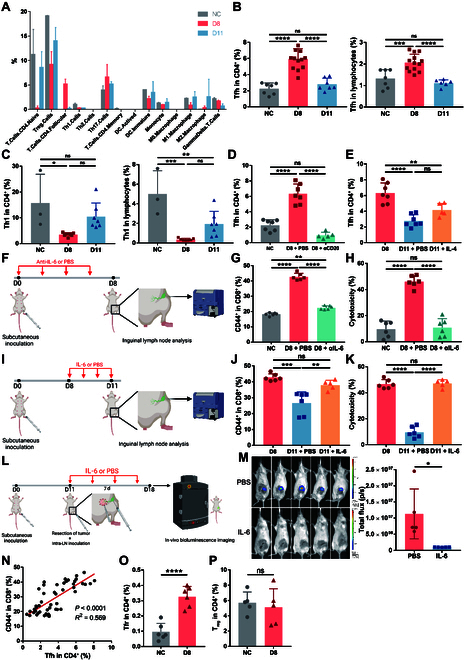
Tfh cells expand and contract along with B cells to directly regulate CD8^+^ T cells. (A) Percentages of immune cells in TDLNs estimated by CIBERSORTx. (B and C) 4T1 cells were injected subcutaneously into the left fourth mammary fat pad of mice. TDLNs were obtained on D8 and D11 for flow cytometry of Tfh cells (B) and Th1 cells (C) (*n* = 7 to 12 mice per group). Inguinal lymph nodes of normal mice were obtained as NC. Among CD4^+^ T cells, Tfh cells were defined as CXCR3^−^CXCR5^+^ and Th1 cells were defined as CXCR3^+^CXCR5^−^. (D) Cryopreserved cells from mice in Fig. 4C were analyzed by flow cytometry (*n* = 5 to 7 mice per group). (E) Cryopreserved cells from mice in Fig. 4E were analyzed by flow cytometry (*n* = 5 to 7 mice per group). (F to H) 4T1 cells were injected subcutaneously into the left fourth mammary fat pad of mice. Starting from D0, anti-IL-6 antibody or PBS was injected subcutaneously 4 times at the inguinal area of each mouse every 2 d (D0, D2, D4, and D6; *n* = 6 mice per group) (F). TDLNs were obtained on D8 for flow cytometry (G) and cytotoxicity assay (H). In cytotoxicity assay, lymph node cells were cocultured with 4T1 tumor cells for 24 h, and cytotoxicity was assessed by LDH release assay. (I to K) 4T1 cells were injected subcutaneously into the left fourth mammary fat pad of mice. Starting from D8, recombinant IL-6 or PBS was injected subcutaneously 4 times at the inguinal area of each mouse daily (D8, D9, D10, and D11; *n* = 5 mice per group) (I). TDLNs were obtained on D11 for flow cytometry (J) and cytotoxicity assay (K). (L and M) 4T1 cells were injected subcutaneously into the left fourth mammary fat pad of mice. On D11, the primary tumor was resected, and 3 × 10^5^ 4T1-luc cells were injected into the subcapsular sinus of TDLN to simulate lymph node metastasis. Starting from D11, recombinant IL-6 or PBS was injected subcutaneously 4 times at the inguinal area of each mouse every 2 d (D11, D13, D15, and D17; *n* = 5 mice per group). Seven days after tumor resection and intra-lymph-node injection, mice were subjected to bioluminescent assay (L). The inguinal flank area was selected to analyze total bioluminescence flux (M). (N) Linear regression and correlation between Tfh cells and CD44^+^CD8^+^ T cells were done with paired data from Fig. 3D, (B), (G), (J), Fig. S7D and E. (O and P) 4T1 cells were injected subcutaneously into the left fourth mammary fat pad of mice. TDLNs were obtained on D8 for flow cytometry of Tfr cells (O) and T_regs_ (P) (*n* = 5 to 6 mice per group). Inguinal lymph nodes of normal mice were obtained as NC. Among CD4^+^ T cells, Tfr cells were defined as CXCR5^+^Foxp3^+^, and T_regs_ were defined as CD25^+^Foxp3^+^. All experiments were repeated at least twice independently. Data are means ± SD. One-way ANOVA tests followed by Tukey’s post hoc test (B to E, G, H, J, and K), unpaired Student’s *t* tests (M, O, and P), and *t* test for Pearson *r* (N) were used to evaluate statistical significance. *P < 0.05; **P < 0.01; ***P < 0.001; ****P < 0.0001.

To figure out the immune compartment with the potency to regulate CD8^+^ T cell activation and also correlated to B cells, we further looked at the subset division results of CIBERSORTx (Fig. 5A). Of note, among CD4^+^ T cells, Tfh cells markedly expanded on D8 and partially reduced on D11, which resembled B cells. Flow cytometry confirmed the trend of Tfh cells revealed by CIBERSORTx (Fig. 5B and Fig. S7A and B). Another CD4^+^ helper T cell subset with the potency to promote CD8^+^ T cell activation, Th1, was ruled out for its contradiction with the alterations of B cells and CD8^+^ T cell activation level (Fig. 5C and Fig. S7A and C).

Tfh cells are a subset of CD4^+^ T cells residing in lymphoid follicles (B cell zone) of lymph nodes and are chemotaxed and expanded upon B cell activation to help with humoral immune response [49–51]. In addition to its important role in facilitating B cells, Tfh cell was recently identified to be necessary for proper functioning of CD8^+^ T cells in lymphoid organs as well [52,53]. Thus, we further tested the possibility of Tfh cells to be the downstream of B cells to regulate CD8^+^ T cell activation in the premetastatic TDLN.

First, Tfh cells were examined in the previous B cell ablation and stimulation settings. B cell ablation led to a lower percentage of Tfh cells close to NC level (Fig. 5D), while IL-4 did not significantly increase the percentage of Tfh cells (Fig. 5E), as IL-4 selectively promotes Th2 differentiation among helper T cells and impedes Tfh cell differentiation [54]. Thus, the level of Tfh cells exactly accorded with CD8^+^ T cell activation status, revealing the potential role of Tfh cells as CD8^+^ T cell regulator. Next, intervention of Tfh cells was carried out by IL-6 antibody neutralization (Fig. 5F) or stimulation (Fig. 5I), as IL-6 is the cytokine crucial for Tfh cell differentiation [51,55]. Successful inhibition and stimulation of Tfh cells were verified by flow cytometry (Fig. S7D and E). Inhibition of Tfh cells on D8 resulted in lower percentages of CD44^+^ activated CD8^+^ T cells (Fig. 5G), while stimulation of Tfh cells on D11 led to a more activated status of CD8^+^ T cells (Fig. 5J). Consistently, cells in TDLNs after inhibition of Tfh cells showed decreased cytotoxicity to 4T1 cells (Fig. 5H), while stimulation of Tfh cells led to increased cytotoxicity (Fig. 5K). The influence of Tfh cells on the antitumor capacity of TDLNs was further tested in vivo (Fig. 5L). Local administration of IL-6 to stimulate Tfh cells in the lymph node could reverse the tumor-tolerant microenvironment in the D11 TDLN and recover its antitumor immunity (Fig. 5M), while direct effects of IL-6 on the primary tumor were ruled out (Fig. S9). Moreover, correlation analysis of CD44^+^CD8^+^ T and Tfh cells showed a positive correlation (Fig. 5N). Overall, Tfh cells underwent expansion and contraction along with B cells and served as the direct regulator of CD8^+^ T cell activation and antitumor immunity.

In immunological researches of vaccination and autoimmunity, it is known that the peak of B cell immune response is followed by spontaneous contraction of germinal centers in lymph nodes to prevent overactivation of humoral immunity [56]. An important mechanism of this negative feedback is through follicular regulatory T (Tfr) that derive from Tfh cells by up-regulation of forkhead box protein P3 (Foxp3) at the peak of activation [57–59]. Since the size of TDLN was determined by B cells, we asked whether Tfr cells also drove the turn of TDLN from enlargement toward contraction in the setting of cancer. By flow cytometry, Foxp3^+^ Tfr cells were found to significantly increase on D8 (Fig. 5O and Fig. S7F and G), supporting the notion that TDLN contracted on D11 due to the spontaneous contraction of germinal centers led by Tfr cells. Considering the impact of B/Tfh cells on CD8^+^ T cell activation, contraction of germinal centers will down-regulate the CD8^+^ T cell activation level and antitumor ability to form the premetastatic microenvironment. To gain a comprehensive understanding about the formation of premetastatic microenvironment in TDLN, we also tested the level of T_regs_ that are responsible for the negative feedback of T cell activation. Unlike Tfr cells, T_regs_ showed no significant difference between NC and D8 (Fig. 5P and Fig. S7H). This further validated that the premetastatic microenvironment on D11 was not induced by the T_reg_-directed self-regulation of T cells but rather by the Tfr-directed self-regulation of B cells.

### IL-21 excreted by Tfh cells is the key regulator of CD8^+^ T cell status in TDLN

To verify the role of Tfh cells as the direct regulator of CD8^+^ T cell activation in the premetastatic TDLN, it still requires clarification how they exert influence on CD8^+^ T cells. Tfh cells carry out their helper T cell functions by excretion of cytokines. According to previous studies [52,60], we speculated that Tfh-derived IL-21 might be the key regulator of CD8^+^ T-cell-direct antitumor immunity in the premetastatic TDLN.

**Fig. 6.**
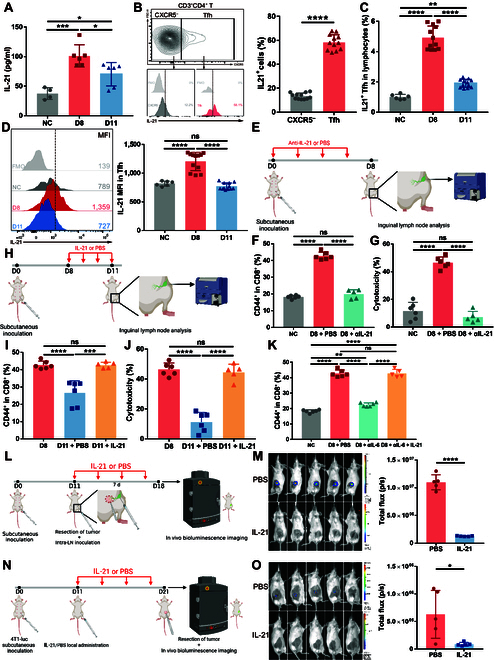
IL-21 excreted by Tfh cells is the key regulator of CD8^+^ T cell status in TDLN. (A) 4T1 cells were injected subcutaneously into the left fourth mammary fat pad of mice. TDLNs were obtained on D8 and D11 for enzyme-linked immunosorbent assay of IL-21 (*n* = 6 mice per group). Inguinal lymph nodes of normal mice were obtained as NC (*n* = 4 mice). (B) TDLNs on D8 were used for flow cytometry analysis of IL-21 in Tfh or CXCR5^−^ non-Tfh cells (*n* = 12 mice). (C and D) Mice were subcutaneously inoculated with 4T1 cells, and lymph nodes were obtained as in (A) (*n* = 12 mice per group). Inguinal lymph nodes of normal mice were obtained as NC (*n* = 6 mice). (E to G) 4T1 cells were injected subcutaneously into the left fourth mammary fat pad of mice. Starting from D0, anti-IL-21 antibody or PBS was injected subcutaneously 4 times at the inguinal area of each mouse every 2 d (D0, D2, D4, and D6; *n* = 5 to 6 mice per group) (E). TDLNs were obtained on D8 for flow cytometry (F) and cytotoxicity assay (G). (H to J) 4T1 cells were injected subcutaneously into the left fourth mammary fat pad of mice. Starting from D8, recombinant IL-21 or PBS was injected subcutaneously 4 times at the inguinal area of each mouse daily (D8, D9, D10, and D11; *n* = 5 to 6 mice per group) (H). TDLNs were obtained on D11 for flow cytometry (I) and cytotoxicity assay (J). (K) 4T1 cells were injected subcutaneously into the left fourth mammary fat pad of mice. Starting from D0, PBS, anti-IL-6 antibody, or anti-IL-6 antibody with IL-21 was injected subcutaneously 4 times at the inguinal area of each mouse every 2 d (D0, D2, D4, and D6; *n* = 5 mice per group). TDLNs were obtained on D8 for flow cytometry. (L and M) 4T1 cells were injected subcutaneously into the left fourth mammary fat pad of mice. On D11, the primary tumor was resected, and 3 × 10^5^ 4T1-luc cells were injected into the subcapsular sinus of TDLN to simulate lymph node metastasis. Starting from D11, recombinant IL-21 or PBS was injected subcutaneously 4 times at the inguinal area of each mouse every 2 d (D11, D13, D15, and D17; *n* = 5 mice per group). Seven days after tumor resection and intra-lymph-node injection, mice were subjected to bioluminescent assay (L). The inguinal flank area was selected to analyze total bioluminescence flux (M). (N and O) 4T1-luc cells were injected subcutaneously into the right fourth mammary fat pad of mice. Starting from D11, recombinant IL-21 or PBS was injected subcutaneously 5 times at the inguinal area of each mouse every 2 d (D11, D13, D15, D17, and D19; *n* = 4 per group). On D21, the primary tumor was resected, and mice were subjected to bioluminescent assay for lymph node metastasis evaluation (N). The inguinal flank area was selected to analyze total bioluminescence flux (O). All experiments were repeated at least twice independently. Data are means ± SD. One-way ANOVA tests followed by Tukey’s post hoc test (A, C, D, F, G, and I to K) and unpaired Student’s *t* tests (B and M) were used to evaluate statistical significance. *P < 0.05; **P < 0.01; ***P < 0.001; ****P < 0.0001.

The intranodal IL-21 levels were first determined by enzyme-linked immunosorbent assay on D8 versus D11. The D8 TDLNs contained much higher levels of IL-21 than NC, while D11 showed a steep decrease, revealing the consistency between IL-21 concentration and CD8^+^ T cell activation level (Fig. 6A). Flow cytometry further helped to determine the main source of IL-21 as Tfh cells, as much higher percentage of IL-21^+^ cells were present in Tfh cells than in CXCR5^−^ non-Tfh cells (Fig. 6B and Fig. S8A). Meanwhile, the percentage of IL-21^+^ Tfh cells (Fig. 6C) and the mean fluorescence intensity (MFI) of IL-21 in Tfh cells (Fig. 6D) were both higher on D8, which could account for the higher intranodal IL-21 level on D8.

To ascertain that IL-21 is the key factor that decides the status of CD8^+^ T cells, we first carried out IL-21 antibody neutralization (Fig. 6E) and stimulation (Fig. 6H). Neutralization of IL-21 on D8 brought the CD8^+^ T cell activation level down to the NC baseline (Fig. 6F), while stimulation with IL-21 on D11 raised the level close to the D8 peak (Fig. 6I). Consistently, cells in TDLN after neutralization of IL-21 showed decreased cytotoxicity to 4T1 cells (Fig. 6G), while IL-21 stimulation led to increased cytotoxicity (Fig. 6J). The proportions of B cells remained unaffected by neutralization/stimulation of IL-21, supporting that IL-21 was an independent factor to determine the antitumor immunity in TDLN (Fig. S8B and C). Furthermore, IL-21 stimulation was able to rescue the influence of Tfh cell inhibition on CD8^+^ T cell activation, which confirmed that IL-21 was the key regulator of CD8^+^ T cells. The decisive impact of IL-21 on the antitumor capacity of TDLNs was further tested in vivo (Fig. 6L). Local administration of IL-21 could reverse the premetastatic microenvironment in the D11 TDLN and completely recover its antitumor immunity (Fig. 6M). The therapeutic effect of IL-21 was further validated in vivo with a spontaneous lymph node metastasis model, which is more clinically relevant (Fig. 6N). IL-21 local administration made TDLNs completely resistant to spontameous metastasis (Fig. 6O), while direct effects of IL-21 on the primary tumor were ruled out (Fig. S9). Therefore, local administration of IL-21 could be a promising procedure to target lymph node metastasis. These results together confirmed that Tfh-derived IL-21 determined the CD8^+^ T cell activation level and antitumor capacity. Thus, the entire mechanism was established about how the size and antitumor immunity of a TDLN synchronously altered before metastasis. B cells expand and contract to determine the size of TDLN and regulate the antitumor immunity of TDLN through the B-Tfh-IL-21-CD8^+^ T cell axis.

### Clinical data validate the central role of B-Tfh-IL-21-CD8^+^ T cell axis in the dynamic alterations of TDLN size and immune status

**Fig. 7.**
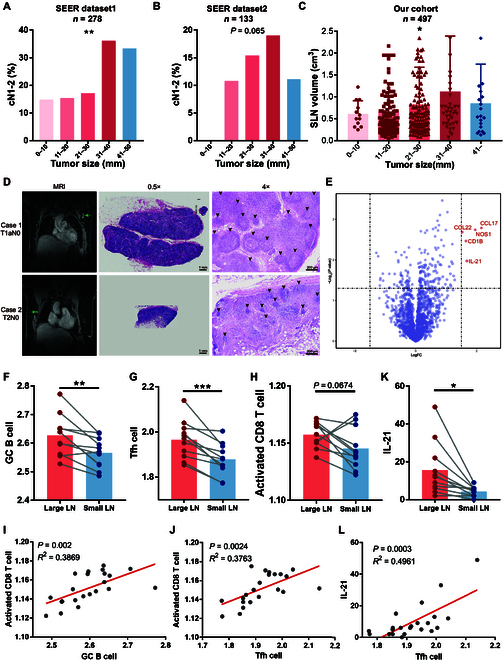
Clinical data validate the central role of B-Tfh-IL-21-CD8^+^ T cell axis in the dynamic alterations of TDLN size and immune status. (A and B) In 2 cohorts from SEER, proportions of cN1-2 among pN0 in patients with different sizes of tumor are shown. (C and D) In our own cohort, SLN volumes in patients with different sizes of tumors were analyzed (C). Magnetic resonance imaging and H&E staining of TDLN were displayed in 2 representative cases (D). (E to L) Analysis of a set of open RNA-seq data. Volcano plot of differentially expressed genes (E), gene expression signature scores of germinal center (GC) B (F), Tfh (G), and activated CD8^+^ T (H) cells are shown. FC, fold change. (I and J) Linear regression and Pearson *r* between germinal center B cell score and activated CD8^+^ T cell score, Tfh cell score, and activated CD8^+^ T cell score, respectively. (K) Paired expression level of IL-21. (L) Linear regression and Pearson *r* between Tfh cell score and IL-21 expression. Data are means ± SD. One-way ANOVA tests (A to C), unpaired Student’s *t* tests (F to H), paired Student’s *t* tests (K), and *t* test for Pearson *r* (I, J, and L) were used to evaluate statistical significance. *P < 0.05; **P < 0.01; ***P < 0.001.

First, we sought to validate the alteration in the size of TDLN clinically. Using the Surveillance, Epidemiology, and End Results (SEER) database, we enrolled patients with T1-2 (tumor < 5 cm) triple-negative breast cancer with pathologically confirmed negative regional lymph nodes (pN0) for analysis. As cN1-2 usually represents presence of enlarged axillary lymph nodes, the proportion of cN1-2 in these pN0 cases can reflect the overall size of lymph nodes. In patients with invasive carcinoma combined with in situ carcinoma components, the relationship between tumor size and the proportion of cN1-2 was analyzed. While the proportion of cN1-2 increased gradually with tumor progression from 0 to 4 cm, it significantly decreased when the tumor grew up to 4 to 5 cm (Fig. 7A). In another group of patients with the entire tumor reported as invasive carcinoma, a similar turning point was observed when the tumor reached 4 to 5 cm (Fig. 7B). To characterize the alteration more precisely, we further used our own breast cancer cohort from 2021 to 2022 for verification. Sentinel lymph node (SLN), which is the first-step TDLN directly influenced by the primary tumor, is used for analysis. Female patients with human epidermal growth factor receptor-2-positive (HER2^+^) and triple-negative invasive breast cancer were included for analysis as both subtypes are of strong immunogenicity. In all, 497 patients with pathologically negative SLNs who did not receive preoperative treatment were enrolled for analysis (Fig. S9). The precise volume of the largest SLN in each case was calculated pathologically. Of these 497 cases, the mean volume of SLN increased gradually with tumor progression from 0 to 4 cm and significantly decreased when the tumor grew up to over 4 cm (Fig. 7C). These results confirmed that there indeed existed an enlargement–contraction dynamic alteration in the TDLNs of patients with breast cancer.

Next, we attempted to explore the corresponding immunological changes behind the changes in size of TDLNs in human breast cancer. Two cases were selected from our cohort, one with small tumor but large SLN and another with large tumor but small SLN. By H&E staining, the large SLN showed distinct dilation of germinal centers, while in the small SLN, there only remained shrunk follicles (Fig. 7D). This provided a preliminary hint that the change in size might be mediated by expansion and contraction of B cells. Further verification of mechanism relied on high-throughput data; hence, all published sequencing data of lymph nodes from patients with breast cancer were collected. Among them, there was only one set of data that provided information on the size of TDLN, which is RNA-seq data of paired large (>15 mm) and small (<5 mm) lymph nodes from patients with triple-negative breast cancer (BioProject: PRJNA658606). Volcano plot of differentially expressed genes revealed the most significantly up-regulated genes in large TDLNs, which were immune-related and contributed to the recruitment and activation of T/B cells, including IL-21 (Fig. 7E). This reflected a more activated immune status in the enlarged TDLNs. To specifically evaluate whether the B-Tfh-IL-21-CD8^+^ T cell axis established in mice also had a role in the size and immune status of human breast cancer, we applied gene expression signature scores of corresponding immune compartments. In line with the findings above, large TDLNs showed higher scores of both germinal center B (activated B) cells (Fig. 7F) and Tfh cells (Fig. 7G). Interestingly, the score of activated CD8^+^ T cells showed a nearly significant difference, which might reflect the up-regulation of CD8^+^ T cell activation level but accompanied by reduction of CD8^+^ T cell proportion as in Fig. 3 (Fig. 7H). Correlation analysis further showed correlations not only between Tfh cells and B cells (Fig. S10A) but also between activated CD8^+^ T cells and both B (Fig. 7I) and Tfh cells (Fig. 7J). When we looked at the expression level of IL-21, significantly higher levels of IL-21 were observed in large lymph nodes (Fig. 7K). The strong correlation between IL-21 and Tfh cells further proved that Tfh cells were the source of IL-21 in TDLN (Fig. 7L). Altogether, these results proved in the clinical setting that the size of TDLN was related to immune status, and these changes are under the regulation of B-Tfh-IL-21-CD8^+^ T cell axis.

## Discussion

Apart from being the most common site of metastasis in breast cancer, TDLN is also the site where antitumor immunity arises. Even so, limited and contradictory knowledge is known about what changes the TDLN goes through before metastasis. In the present study, we discovered an enlarged-contracted dynamic alteration in the size of TDLN, which coexisted with an activated-inhibited alteration in immune status. Mechanistically, the alteration of TDLN in size and antitumor immunity was determined by the B-Tfh-IL-21-CD8^+^ T cell axis. On the basis of the mechanism, some TDLN-targeting measures to reinforce their antitumor immunity were raised and verified. Thus, our results shed light on what interventions of TDLNs should be taken on the basis of their size and offer some mechanistic insights into how the TDLNs can be boosted to resist metastasis.

The size of TDLNs from clinical examinations is an important information to evaluate whether the lymph node might be metastatic and should be surgically removed. Different from the premetastatic microenvironments in other organs, we discovered in TDLNs a unique enlarged phase before metastasis, and this enlargement represented an immune-activated status completely resistant to metastasis. The phenomenon was further verified in multiple clinical settings including our own cohort and open datasets. Since the antitumor immunity and memory generated in TDLNs are known to be crucial for favorable survival and response to adjuvant therapies [32–34,61], excision of these enlarged activated lymph nodes is not only unnecessary but also eliminating the source of systemic antitumor immune response. Therefore, our findings should raise the importance of pathological examination of enlarged TDLNs by fine needle aspiration instead of direct lymph node dissection, since preservation of uninvolved activated lymph nodes is of remarkable benefits whether with or without after treatments.

After the unique activated state, the TDLN turned into an immunosuppressed state that favored metastasis like in other organs. Mechanistically, the antitumor immunity mediated by CD8^+^ T cells was determined by IL-21-secreting Tfh cells in TDLNs, and the turn from activation to suppression was driven by the self-regulation of Tfh cells. Thus, our findings indicate that Tfh cells and IL-21 are important targets to boost the immune activity in TDLNs to make them intrinsically resistant to metastasis. On the basis of the mechanistic findings, we have verified 2 targeting strategies in the present study and found that local administration of IL-6 or IL-21 in the TDLN draining area could completely recover the activation level and antimetastasis ability of TDLNs. These findings provided important evidence that by targeting IL-21-secreting Tfh cells or directly administrating IL-21, the metastasis-supportive microenvironment in premetastatic TDLNs could be reversed, and even existing metastasis in lymph nodes could be eliminated by immunity. Although further clinical verification is still needed, our findings have offered some new thoughts for the intervention of TDLNs in breast cancer. Because of the dynamic change of TDLNs, different therapeutic strategies to inhibit lymph node metastasis should be adopted on the basis of the different status of TDLNs.

In terms of mechanisms in this study, we have confirmed the key role of B-Tfh-IL-21-CD8^+^ T cell axis in the regulation of TDLNs. B cells in TDLNs were previously reported to be prometastatic by secreting pathologic immunoglobulin to increase chemotaxis of tumor cells [13]. In this study, however, we focused on the immune microenvironment in TDLNs regulated by Tfh cells, and B cells are only the determinant of size and upstream of Tfh cells. Our findings are not contradictory but rather supplementary to the direct negative effects of B cells on tumor cells. Tfh cells have also been reported in recent years to help with CD8^+^ T cell activation in microbial infection and vaccination. Here, we uncovered their new role to determine the changes in size and immune status of premetastatic TDLNs. Moreover, we confirmed that the Tfh-induced CD8^+^ T cell activation was tumor-specific, so the early-stage enlarged or interleukin-treated TDLNs possessed the ability to resist metastasis from the primary tumor. Overall, our discoveries in mechanism expand the existing theories and may benefit our understanding of TDLNs.

Despite our efforts to give an overall perspective, limitations still exist in this study. First, the specific mechanism by which breast cancer selectively activates B cells is lacking. Many other studies have already reported that B cells are selectively activated by breast cancer especially in lymph nodes [45–48], and our findings again confirmed the phenomenon. Future efforts to establish the mechanistic link between breast cancer and B cell activation may help better understanding the biology and immunogenicity of breast cancer. Second, whether the same alterations also exist in premetastatic TDLNs of other tumor types needs further investigation. In our opinion, the dynamic change and the B-Tfh-IL-21-CD8^+^ T cell axis may not be common in other tumors because of the unique nature of breast cancer to activate B cells. In contrast to breast cancer that was formerly described as “cold tumor” for its inability to induce T cell expansion, other “hot tumors” such as melanoma can effectively boost T cell expansion and may consequently generate totally different changes in their premetastatic TDLNs.

## Methods

### Study design

The goal of this study was to elucidate the dynamic alteration in the size and immune status of TDLNs before metastasis in breast cancer, and the key regulatory mechanism of the dynamic alteration, so as to seek strategies to target lymph node metastasis. First, we observed the dynamic alteration in the size and immunity of premetastatic TDLNs. Second, we investigated the regulatory mechanism behind the dynamic alteration. Next, on the basis of the mechanism, we tested some TDLN-targeting measures to reinforce their antitumor immunity. Last, we did clinical validations of the phenomena and mechanism. Data from both animal models and patients with breast cancer were adopted for investigation. Data inclusion/exclusion criteria in the clinical validation with SEER were detailed in Fig. S9. For in vivo observation of the dynamic alteration, 3 independent models were adopted. In general, at least 2 independent experiments were conducted with technical replicates. All sample sizes are indicated in figure legends. For clinical validations, multiple sources of data were covered, including large-scale database, our own cohort, and RNA-seq data.

### Mice

Six- to 8-week-old female BALB/c mice were purchased from Charles River. All mice were maintained under specific-pathogen-free conditions in the Animal Core Facility of Nanjing Medical University. All animal experiments were following the protocols (IACUC-2202022) reviewed and approved by the Institutional Animal Care and Use Committee (IACUC), Animal Core Facility of Nanjing Medical University. All animals were randomly assigned to different experimental groups.

### Cell lines and cell culture

The BALB/c mouse-derived mammary carcinoma 4T1 and EMT6 cell lines were purchased from the Chinese Academy of Sciences (China). 4T1-luc cell line was generated by transfecting luciferase-expressing lentivirus LV16 (U6/Luciferase17&Puro) into 4T1 cells. Briefly, 5 to 10 multiplicity of infection of recombinant lentivirus (GenePharma, China) was mixed in serum-free medium with polybrene (6 to 8 μg/ml) and then used to incubate 4T1 cell line at 37 °C for 24 h. Puromycin was applied to select transfected 4T1 tumor cells at a concentration of 2 μg/ml in the complete medium. The selected 4T1-luc tumor cell line after transfection was incubated in puromycin-containing medium for another 7 d. The 4T1 tumor cells were validated for the efficiency of transfection by Luciferase Assay System (Promega, China). 4T1, EMT6, and 4T1-luc cell lines were cultured in Dulbecco’s modified Eagle’s medium (HyClone, USA) supplemented with penicillin (100 U/ml), streptomycin (100 mg/ml; HyClone, USA), and 10% (v/v) fetal bovine serum (FBS; HyClone, USA). All cells were cultured in a 37 °C incubator with 5% CO_2_. Mycoplasma was confirmed negative by the standard polymerase chain reaction method.

### Tumor models

In the subcutaneous tumor models, 4T1 tumor cells were injected subcutaneously into the left second or fourth mammary fat pad at a density of 5 × 10^5^ cells in 100 μl of phosphate-buffered saline (PBS; HyClone, USA). In the intraductal tumor model, 2 × 10^4^ 4T1 tumor cells in 20 μl of PBS were injected through the nipple into the mammary duct of the left second mammary gland under a stereoscope using a 50-μl microliter syringe with a 33-gauge metal hub needle (Hamilton, Switzerland). Trypan blue (0.2%) was contained in PBS to show successful injection into the mammary ductal tree. Lymph node size was measured by calipers. Lymph node volume (in cubic millimeters) was calculated by the following formula: *V* = *π*/6 × *L* × *H*^2^, where *H* is the shorter diameter and *L* is the longer diameter. For detection of metastasis, slices of lymph nodes were obtained every 0.2 mm and then subjected to H&E staining and microscopic examination for tumor cell clusters. The tumor size did not reach 1.5 cm in diameter when the mice were euthanized.

### TDLN bulk RNA-seq library construction

Total RNA was extracted from frozen TDLNs using TRIzol reagent (Thermo Fisher Scientific, USA) following the standard procedure. The purity and quantity of the total RNA were evaluated by Bioanalyzer 2100 and RNA 6000 Nano LabChip Kit (Agilent, USA). Samples with RNA integrity number (RIN) number of >7.0 were used for further analysis. Dynabeads oligo(dT) (Thermo Fisher Scientific, USA) were used to obtain purified polyadenylate mRNA from total RNA (1 μg), and the mRNA was purified for 2 rounds. Then, the mRNA was fragmented into small pieces under elevated temperatures by divalent cations. According to the protocol for the mRNA-seq sample preparation kit (Illumina, USA), the cleaved RNA fragments were reverse-transcribed to generate the ultimate cDNA library. The average insert size for the final cDNA library was 300 base pairs (bp) (±50 bp). The paired-end sequencing was performed on an Illumina sequence platform (LC Science, Hangzhou, China).

### Bulk RNA-seq data analysis

For quality control, the sequence was verified by fastp software (https://github.com/OpenGene/fastp). HISAT2 (https://ccb.jhu.edu/software/hisat2) was used to map reads to the *Mus musculus*. StringTie (https://ccb.jhu.edu/software/stringtie) was used to assemble the mapped reads of each sample. To estimate the expression levels of all transcripts, StringTie and ballgown (http://www.bioconductor.org/packages/release/bioc/html/ballgown.html) were used after the final transcriptome was generated. FPKM (fragment per kilobase of transcript per million mapped reads) value was calculated by StringTie and ballgown to perform mRNAs expression abundance.

### Immune cell compositions analysis

For immune cell composition prediction in TDLNs, CIBERSORTx (https://cibersortx.stanford.edu/) was used to analyze standard RNA-seq expression quantification metrics. The reference marker gene expression profile matrix was assigned with a widely used mouse immune cell signature matrix (28084418). During the analysis, quantile normalization was disabled, and 1,000 permutations were used to calculate *P* values.

### Differentially expressed genes and gene ontology enrichment analysis

We determined the differentially expressed genes of RNA-seq by R package limma (v3.48.3). Differentially expressed genes were selected with log(fold change) higher than 2 or lower than –2 and statistical significance (*P* < 0.05). Differentially expressed genes were then subjected to enrichment analysis of gene ontology (GO) functions by R package clusterProfiler (v 4.0.5).

### Lymph node metastasis model

The primary tumor was first resected, and the location of the incision was about 0.5 cm above the hip joint of the mice. After resection of the tumor, 1 × 10^5^ or 3 × 10^5^ 4T1-luc cells in 3 μl of PBS were injected through the incision into the subcapsular sinus of the left inguinal lymph node under a stereoscope using a 50-μl syringe with a 33-gauge metal hub needle (Hamilton, Switzerland). Trypan blue (0.2%) were contained in PBS to show successful injection into the lymph node and exclude leakage into the fat pad. The wound was sutured with 4/0 nylon. All procedures in vivo were aseptic.

### Bioluminescent assay

d-Luciferin (GoldBio, USA) was dissolved in Dulbecco’s PBS (HyClone, USA) without Ca^2+^ or Mg^2+^ to a concentration of 15 mg/ml. The mice were injected intraperitoneally with 10 μl of d-luciferin solution per gram of body weight. Kinetic curve was generated to determine the peak signal time for lymph node imaging 10 min after injection. Mice for experiments were anesthetized with isoflurane and all photographed in IVIS Spectrum (PerkinElmer, USA) 10 min after luciferin injection. The inguinal flank area was selected for analyzing the total bioluminescence flux.

### CD8^+^ T cell depletion

Starting from 1 day before tumor resection and intra-lymph-node injection (D7 or D10), each mouse was injected intraperitoneally 4 times with 200 μg of anti-CD8a (clone: 53-6.7, BioLegend, USA) every 2 d.

### Preparation of single-cell suspension from TDLN

After mice were euthanized, the TDLNs were collected. The harvested TDLNs were then crushed gently with the plunger of a 10-ml syringe and passed through a 70-μm nylon mesh cell strainer (Biosharp, China). Single cells of TDLN were resuspended with PBS for further analysis or with 10% dimethyl sulfoxide in FBS for cryopreservation.

### Flow cytometry

For flow cytometry, single cells of TDLN were used. For surface staining of cells, cells were stained with fluorescence-conjugated antibodies in flow cytometry staining buffer (BioLegend, USA) for 30 min at 4 °C. Surface-stained cells were fixed by fixation buffer (BioLegend, USA) for intracellular protein staining. Then, the cells were permeabilized by intracellular staining permeabilization wash buffer (BioLegend, USA) following the manufacturer’s protocol. Permeabilized cells were inoculated with staining antibodies in a permeabilization wash buffer to stain intracellular protein. The following fluorescence-conjugated antibodies were used: fluorescein isothiocyanate anti-mouse CD3 antibody (BioLegend, catalog no. 100204), APC/Fire 750 anti-mouse CD4 antibody (BioLegend, catalog no. 100460), Brilliant Violet 421 anti-mouse CD4 antibody (catalog no. 100443), APC anti-mouse CD8a antibody (catalog no. 100711), Pacific Blue anti-mouse CD8a antibody (catalog no. 100725), phycoerythrin (PE) anti-mouse/human CD44 antibody (catalog no. 103007), APC/Cyanine7 anti-mouse CD69 antibody (catalog no. 104525), APC anti-mouse CD25 antibody (catalog no. 102011), PE anti-mouse Foxp3 antibody (catalog no. 126404), PE anti-mouse CD183 (CXCR3) antibody (catalog no. 126505), APC anti-mouse CD185 (CXCR5) antibody (catalog no. 145506), and PE anti-mouse IFNγ antibody (catalog no. 505807). Cell data were acquired on the BD FACSCanto cytometer (BD Biosciences) and were analyzed using FlowJo 10.8.1 software.

### Synthetic peptide

H-2Ld-restricted peptide AH1_6-14_ (SPSYVYHQF) was synthesized by GenScript Corporation (Nanjing, China). The purity of the peptides was 98.5%, which was validated by high-performance liquid chromatography. H-2Kb-restricted peptide OVA_257–264_ (SIINFEKL) was synthesized by Genescript Corporation (Nanjing, China). The purity of the peptides was 95.9%, which was validated by high-performance liquid chromatography. These peptides were then used for tumor-specific T cell analysis.

### Analysis of tumor-specific T cells

Lymph node cells were resuspended at 2 × 10^6^ cells/ml in RPMI 1640 medium. Then, the splenocytes (5 × 10^5^) were cocultured with peptide (10 μg/ml) for 12 h. Protein transport inhibitor cocktail (eBioscience, USA) was added in the last 4 h to enhance intracellular cytokine staining. Analysis of intracellular IFNγ production was evaluated by flow cytometry.

### In vitro specific cytotoxicity assay

4T1 or EMT6 cell lines were seeded at a density of 5 × 10^3^ per well in 96-well plates 24 h before coculture. Lymph node cells and the tumor cells were cocultured at the ratio of 100:1 in RPMI 1640 medium (HyClone, USA) supplemented with penicillin (100 U/ml), streptomycin (100 μg/ml), and 5% FBS and recombinant mouse IL--2 (40 ng/ml; BioLegend, USA). After 24 h of coculture, the cytotoxicity was assessed using the Cytotoxicity LDH (lactate dehydrogenase) Assay Kit (Dojindo, Japan).

### B cell depletion

Starting from D0 after tumor inoculation, each mouse was injected subcutaneously 4 times at the inguinal area with 50 μg of anti-CD20 (BioLegend, USA) or PBS as control every 2 d.

### In vivo interleukin stimulation assay

Starting from D8 after tumor inoculation, each mouse was injected subcutaneously 4 times at the inguinal area with 25 μg of recombinant IL-4, IL-6, or IL-21 (BioLegend, USA) daily (D8, D9, D10, and D11). In parallel, mice injected with PBS in the same way were set as control.

### In vivo interleukin neutralization assay

Starting from D0 after tumor inoculation, each mouse was injected subcutaneously 4 times at the inguinal area with 25 μg of anti-IL-6 or anti-IL-21 antibody (BioLegend, USA) every 2 d (D0, D2, D4, and D6). In parallel, mice injected with PBS in the same way were set as control.

### Statistics

Medians and interquartile ranges or mean and SD were used to describe continuous variables mentioned in the study. Proportions and frequencies were used to describe the categorical variables mentioned.

The paired or unpaired *t* test and Wilcoxon rank sum test were applied in RNA-seq analysis to compare continuous variables. Pearson’s chi-square and Fisher’s exact tests were used to compare categorical variables. All statistics from high-throughput experiments were analyzed using R (v 4.1.1, https://www.r-project.org/).

For data from experiments in vitro or in vivo, 2-tailed *t* tests were used for all comparisons between 2 groups. Ordinary one-way analysis of variance (ANOVA) with Tukey’s multiple comparisons test was utilized to compare various groups.

## Data Availability

The datasets generated during and/or analyzed during the current study are available from the corresponding author on reasonable request.
