# Supplementary material for: B Cells and IL-21-Producing Follicular Helper T Cells Cooperate to Determine the Dynamic Alterations of Premetastatic Tumor Draining Lymph Nodes of Breast Cancer
Source: Research (Wash D C). 2024 Mar 29;7:0346. doi: 10.34133/research.0346 (PMC10981934; doi:10.34133/research.0346)
Supplement: Supplementary 1 — Figs. S1 to S11 [file research.0346.f1.zip › Revised Supplementary Information.docx]

**Supplementary Figures: Figure S1-S10**


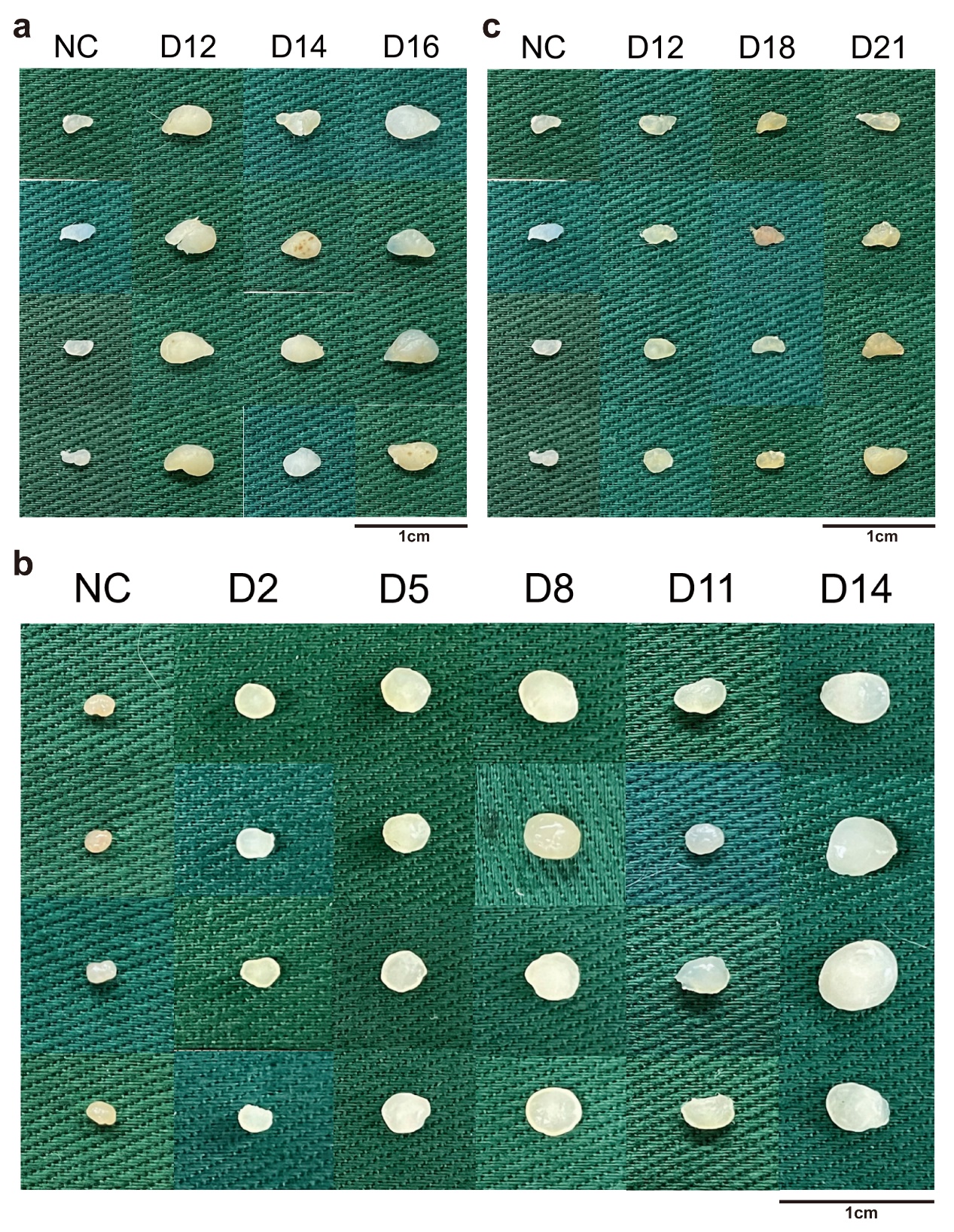


**Figure S1. TDLNs of breast cancer undergo enlargement and subsequent contraction prior to metastasis.** (**a**) Representative photos of TDLNs at different timepoints in the 2# subcutaneous model. (**b**) Representative photos of TDLNs at different timepoints in the 4# subcutaneous model. (**c**) Representative photos of TDLNs at different timepoints in the 2# intraductal model. All photos are cut into 1×1cm squares according to rulers included in each photo and adjusted to the same size for direct comparison.


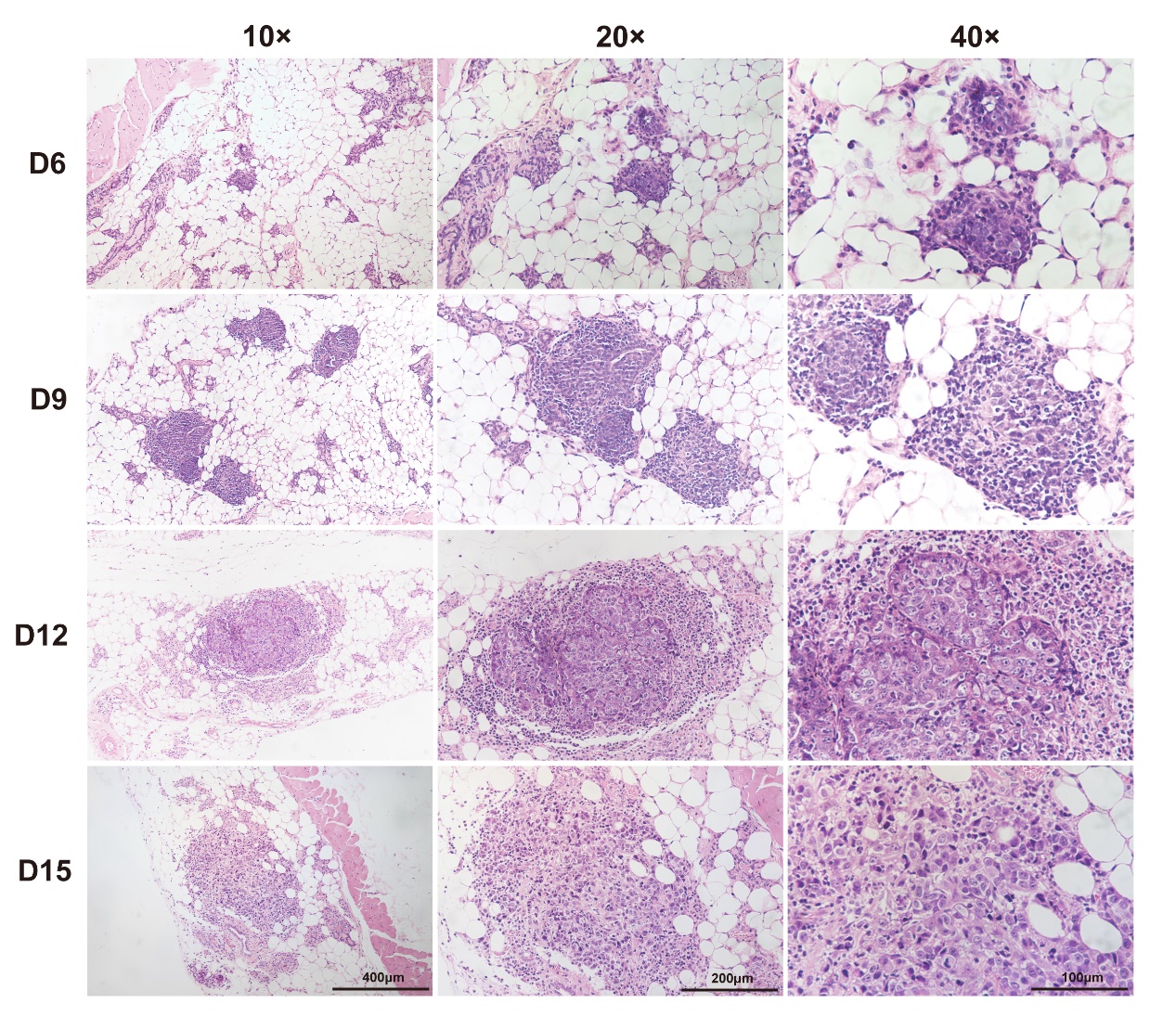


**Figure S2. TDLNs of breast cancer undergo enlargement and subsequent contraction prior to metastasis.** Representative H&E staining of murine breast glands inoculated with 4T1 cells at different timepoints in the 2# intraductal model.


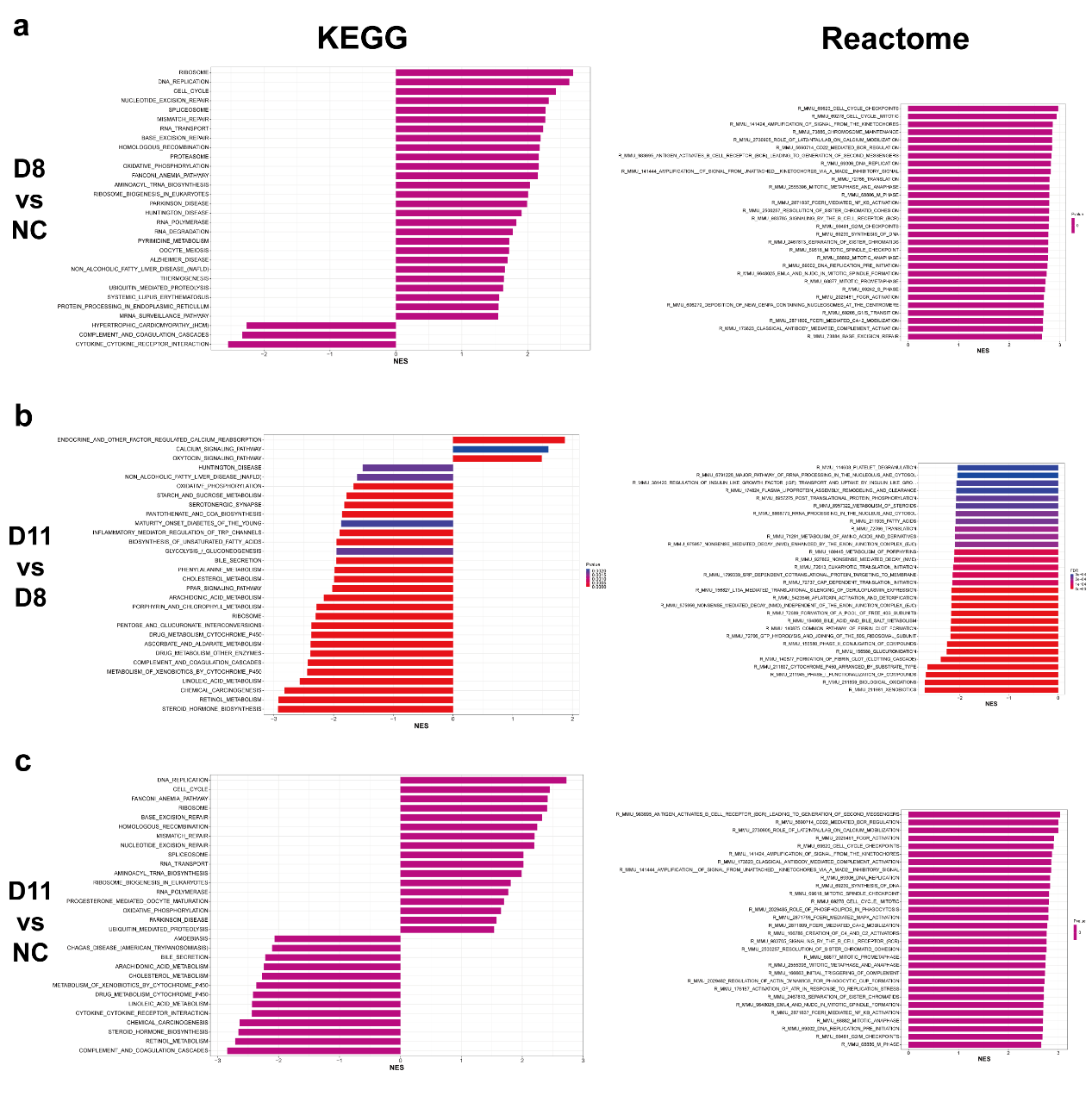


**Figure S3. The antitumor ability of TDLN is parallel to its dynamic alteration in size.** (**a**-**c**) Mice were treated as in (Figure 2a,c). KEGG and reactome pathways enriched in differentially expressed genes of mice’s TDLNs are shown between D8 and NC (a), D11 and D8 (b), D11 and NC (c) respectively. The y-axis shows the top 30 enriched categories, with up-regulated pathways pointing positive to the right and down-regulated pathways pointing negative to the left.


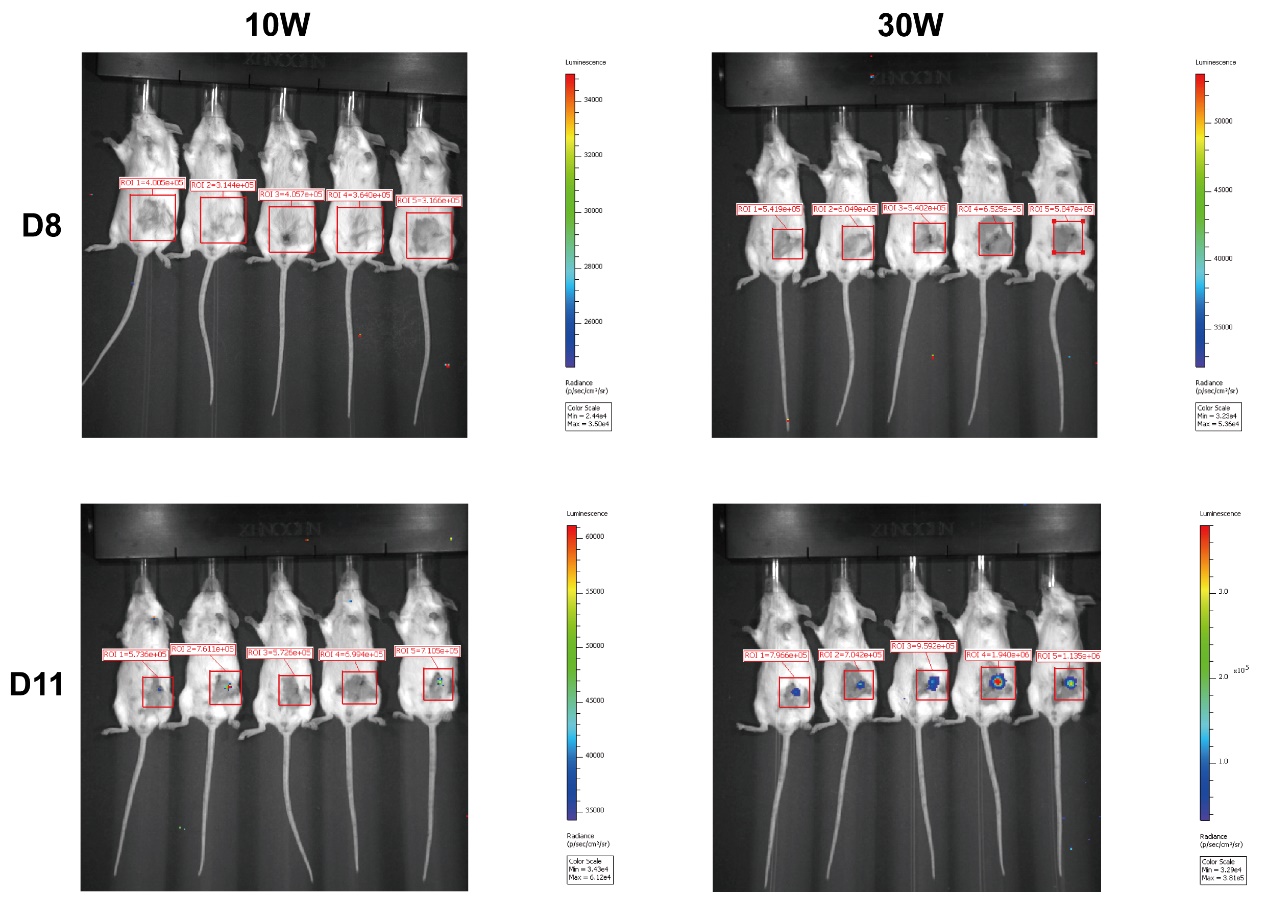


**Figure S4. The antitumor ability of TDLN is parallel to its dynamic alteration in size.** The selected inguinal flank area for analyzing the total bioluminescence flux, with the total flux value marked on each mouse.


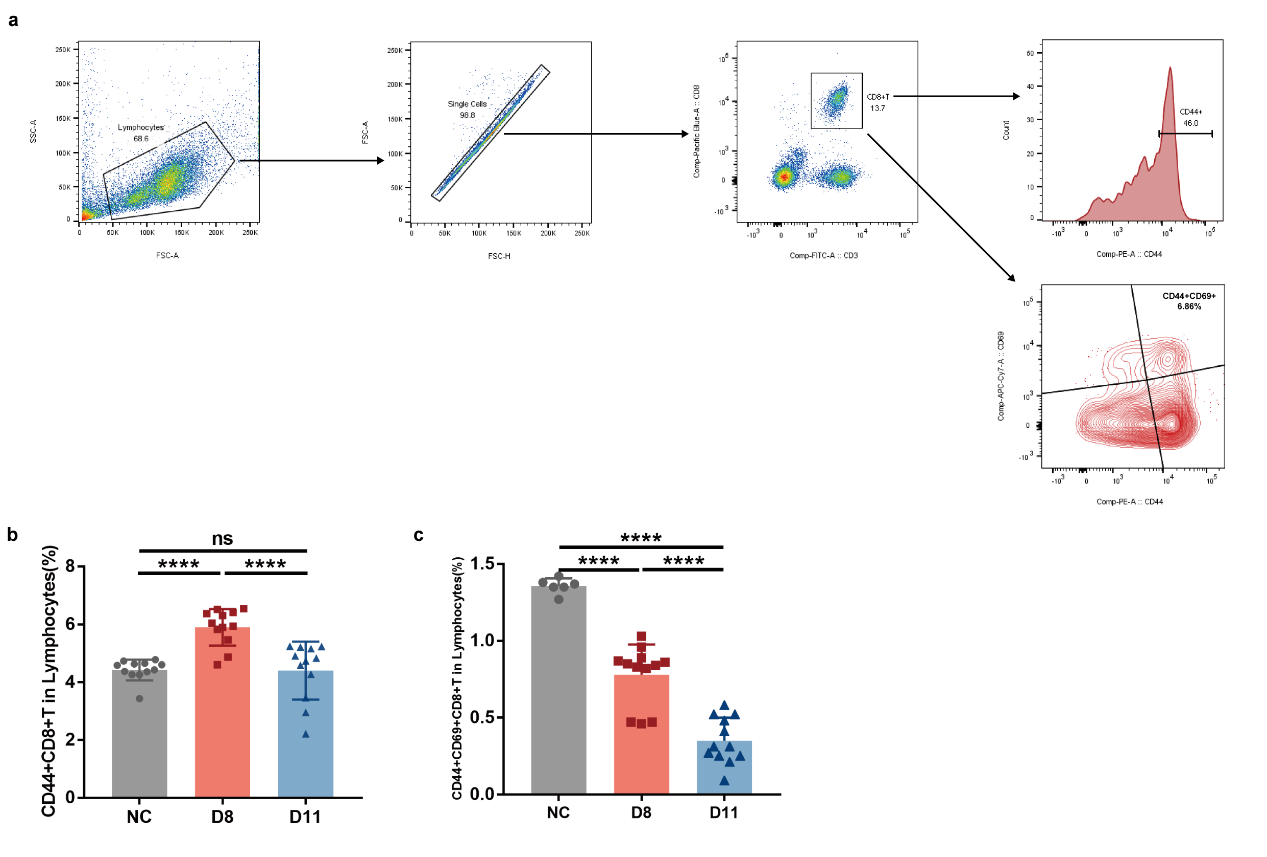


**Figure S5. The changing antitumor ability of TDLN is mediated by tumor-specific CD8+T cells.** (**a**) Gating strategy for analysis of CD44+ and CD44+CD69+ cells in CD8+T cells. (**b**, **c**) Mice were treated as in (Figure 4d-f). The proportion of CD44+cells (b) and CD44CD69+cells (c) in lymphocytes were shown. Data are mean ± SD. One-way ANOVA tests followed by Tukey’s post hoc test was used to evaluate statistical significance. *P < 0.05; **P < 0.01; ***P < 0.001; ****P < 0.0001.


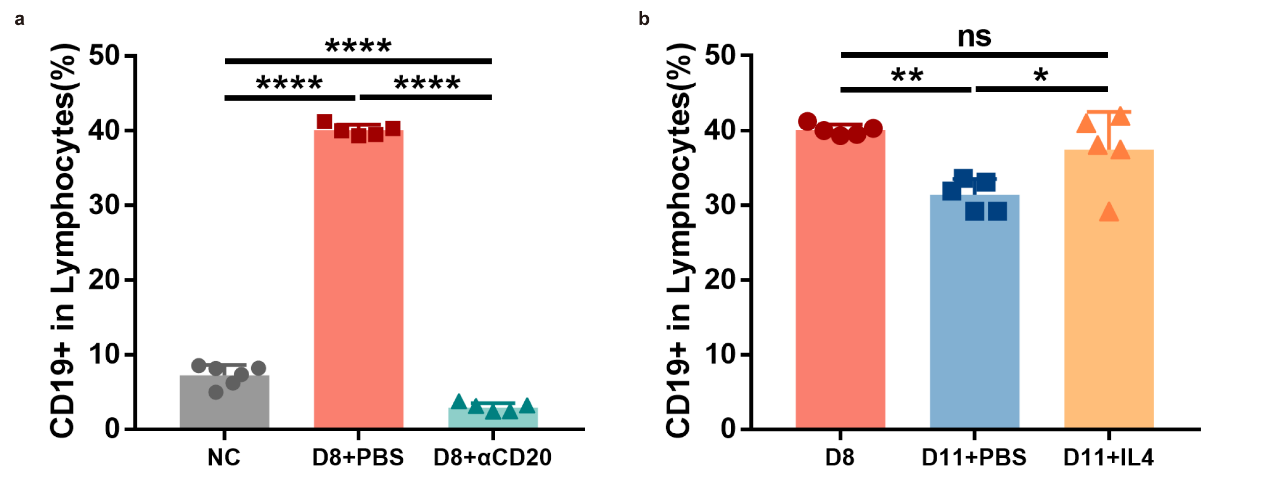


**Figure S6. Expansion and contraction of B cells determine the size of TDLN and regulate CD8+T cell activation.** (**a**) Mice were treated as in (Figure 4c). The proportions of CD19+B cells in lymphocytes were shown (n=5 to 6 mice per group). (**b**) Mice were treated as in (Figure 4d). The proportions of CD19+B cells in lymphocytes were shown (n=5 mice per group). Data are mean ± SD. One-way ANOVA tests followed by Tukey’s post hoc test was used to evaluate statistical significance. *P < 0.05; **P < 0.01; ***P < 0.001; ****P < 0.0001.


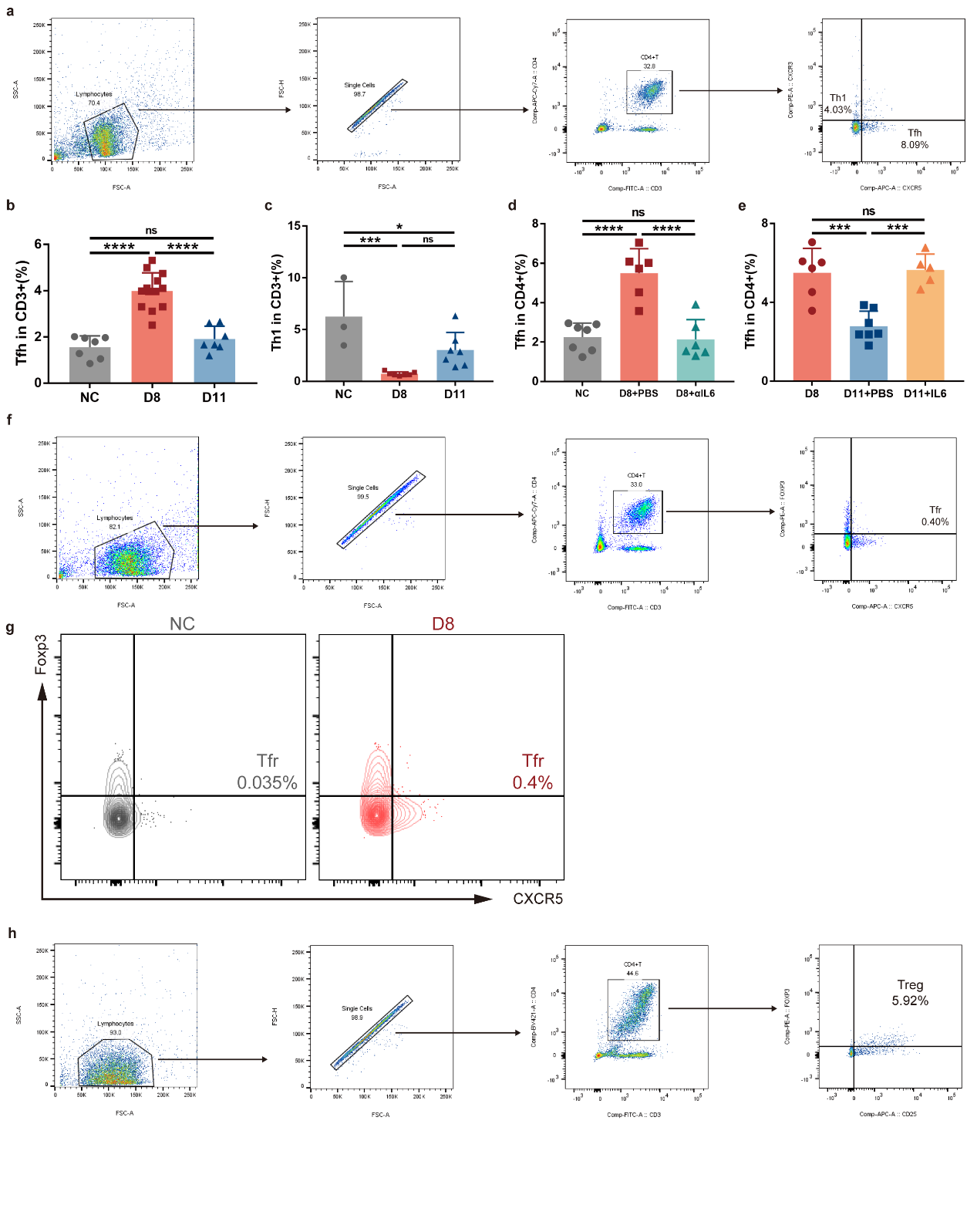


**Figure S7. Follicular helper T cells expand and contract along with B cells to directly regulate CD8+T cells.** (**a**) Gating strategies for Tfh and Th1 cells. (**b**, **c**) Mice were treated as in (Figure 5b,c). The proportions of Tfh (B) and Th1 (C) cells in CD3+T cells were shown. (**d**, **e**) Successful inhibition (d) and stimulation (e) of Tfh cells were verified by flowcytometry. (**f**) Gating strategy for Tfr cells. (**g**) Representative flow plots of Tfr cells in 5M. (**h**) Gating strategy for Treg cells. Data are mean ± SD. One-way ANOVA tests followed by Tukey’s post hoc test was used to evaluate statistical significance. *P < 0.05; **P < 0.01; ***P < 0.001; ****P < 0.0001.


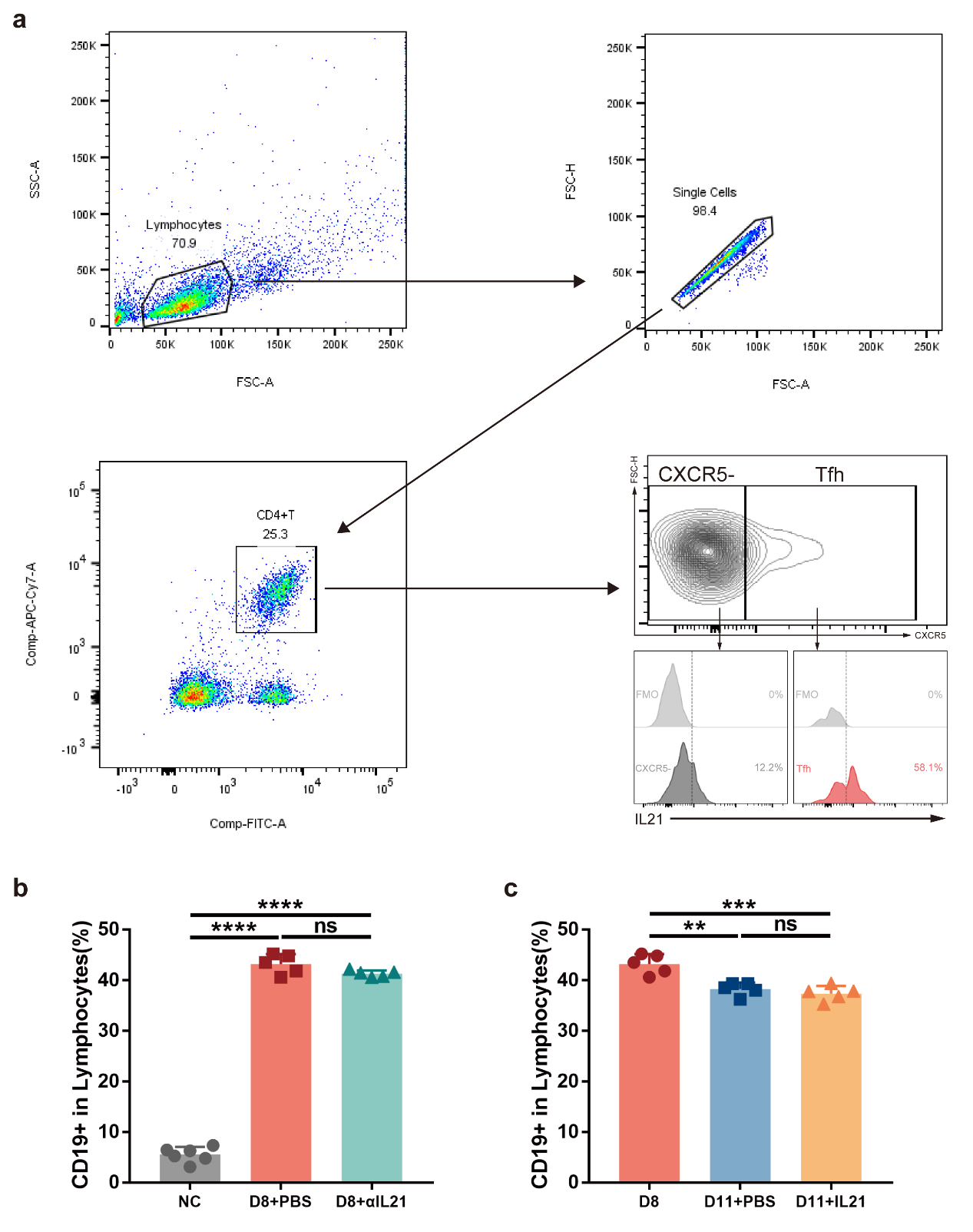


**Figure S8. IL21 excreted by Tfh cells is the key regulator of CD8+T cell status in TDLN.** (**a**) Gating strategy for analysis of IL21 in Tfh or non-Tfh cells in. (**b**, **c**) B cells were examined by flowcytometry after IL21 inhibition (b) and stimulation (c). Data are mean ± SD. One-way ANOVA tests followed by Tukey’s post hoc test was used to evaluate statistical significance. *P < 0.05; **P < 0.01; ***P < 0.001; ****P < 0.0001.


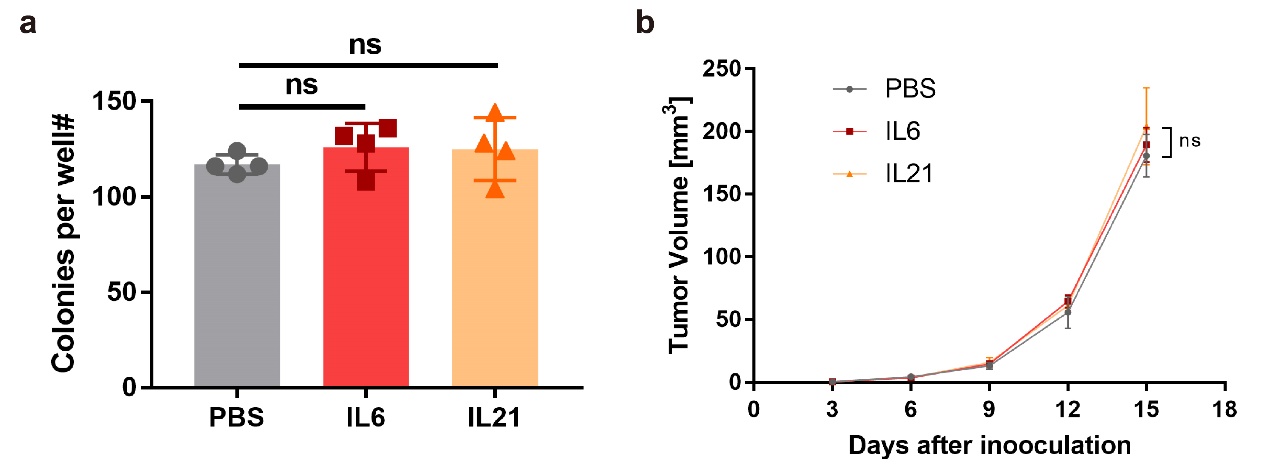


**Figure S9. IL6/IL21 have no direct influence on tumor cells.** (**a**) Colony formation assays after addition of IL6 or IL21 versus normal complete medium. **(b**) Tumor growth curves after local administration of IL6 or IL21 versus PBS.


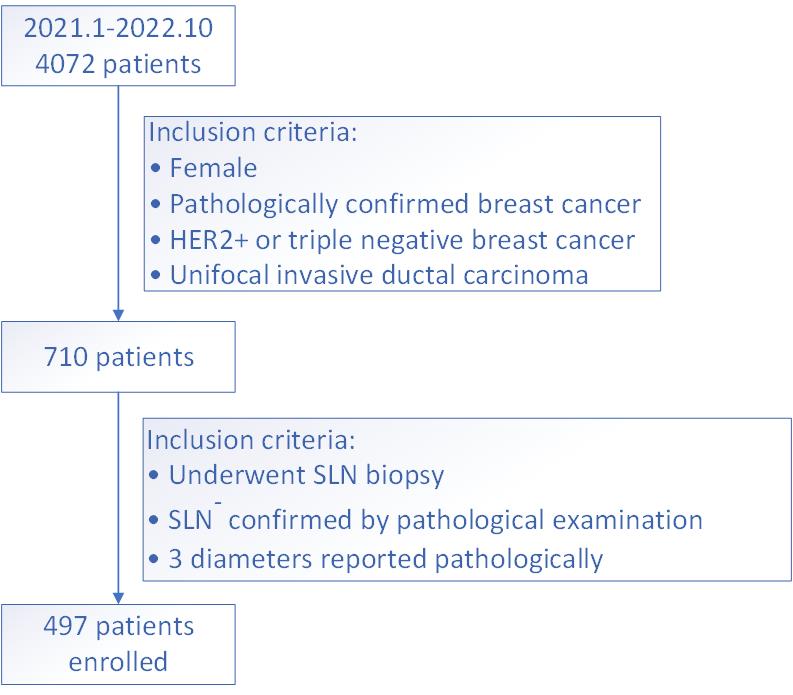


**Figure S9. Clinical data validate the central role of B-Tfh-IL21-CD8+T cell axis in the dynamic alterations of TDLN size and immune status.** To enroll patients from our own cohort for analysis, inclusion and exclusion criteria were as shown.

**
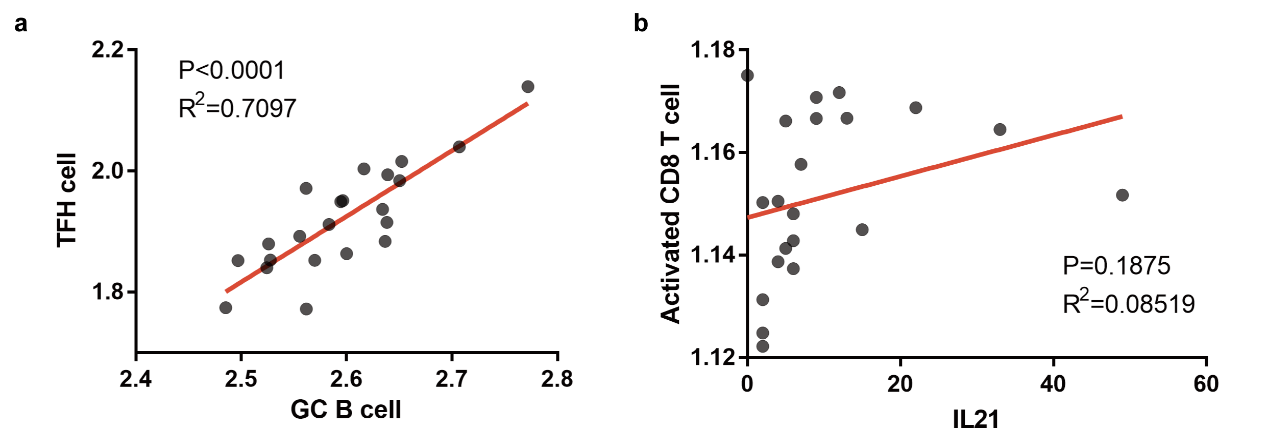
**

**Figure S10. Clinical data validate the central role of B-Tfh-IL21-CD8+T cell axis in the dynamic alterations of TDLN size and immune status.** Using the published RNA-seq data of TDLNs, Linear regression and Pearson r were shown between the score of Tfh cells and the score of GC B cells (**a**), IL21 expression and the score of activated CD8+T cells (**b**). T test for Pearson r was used to evaluate statistical significance.
