# Supplementary figures and images for: B Cells and IL-21-Producing Follicular Helper T Cells Cooperate to Determine the Dynamic Alterations of Premetastatic Tumor Draining Lymph Nodes of Breast Cancer
Source: Research (Wash D C). 2024 Mar 29;7:0346. doi: 10.34133/research.0346 (PMC10981934; doi:10.34133/research.0346)

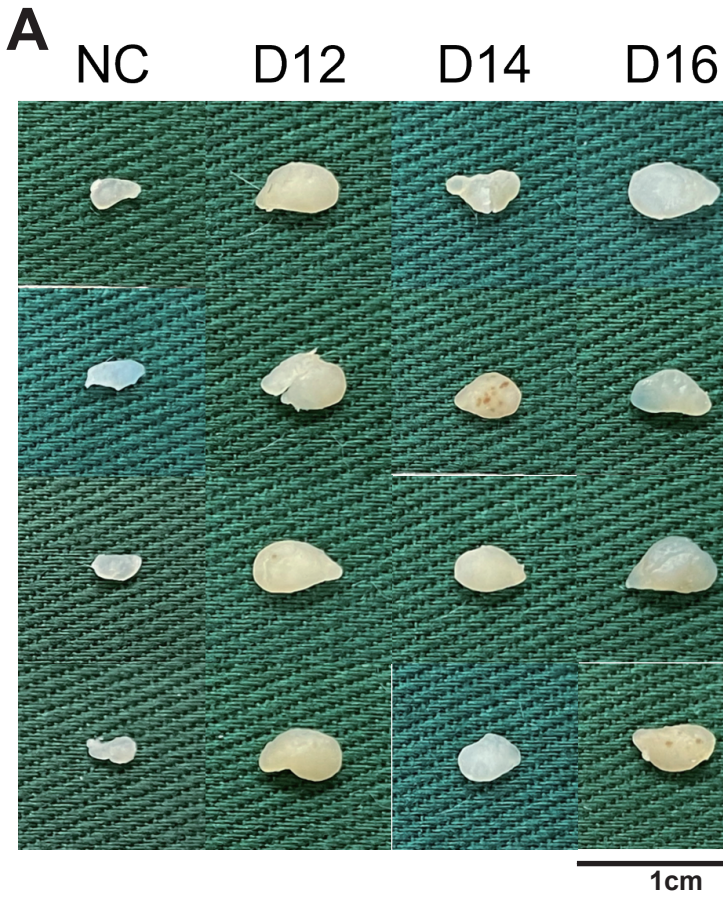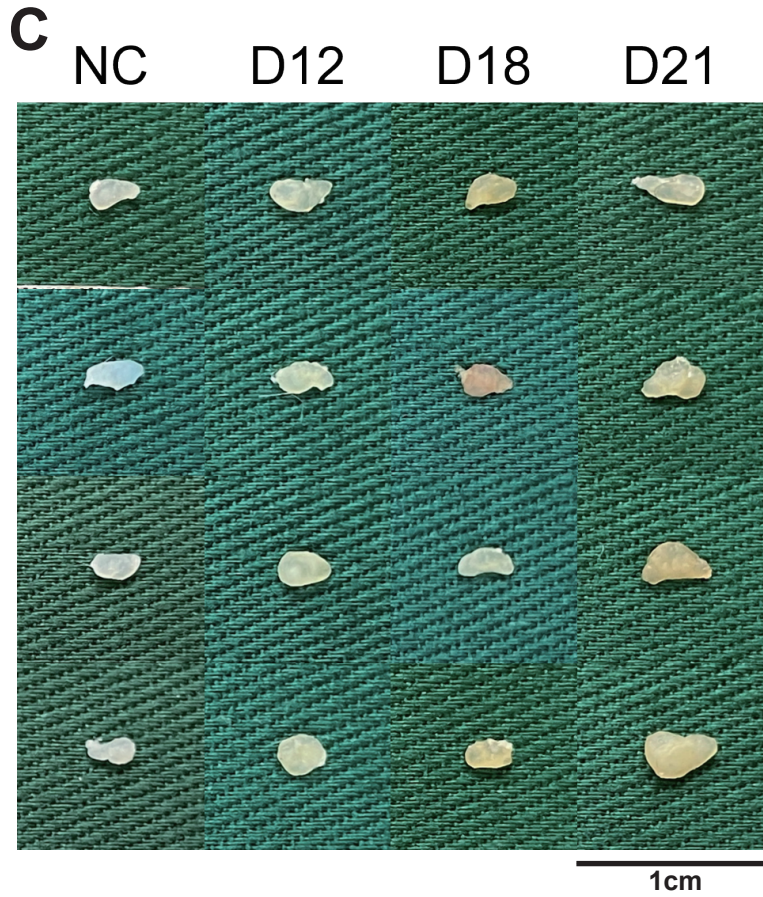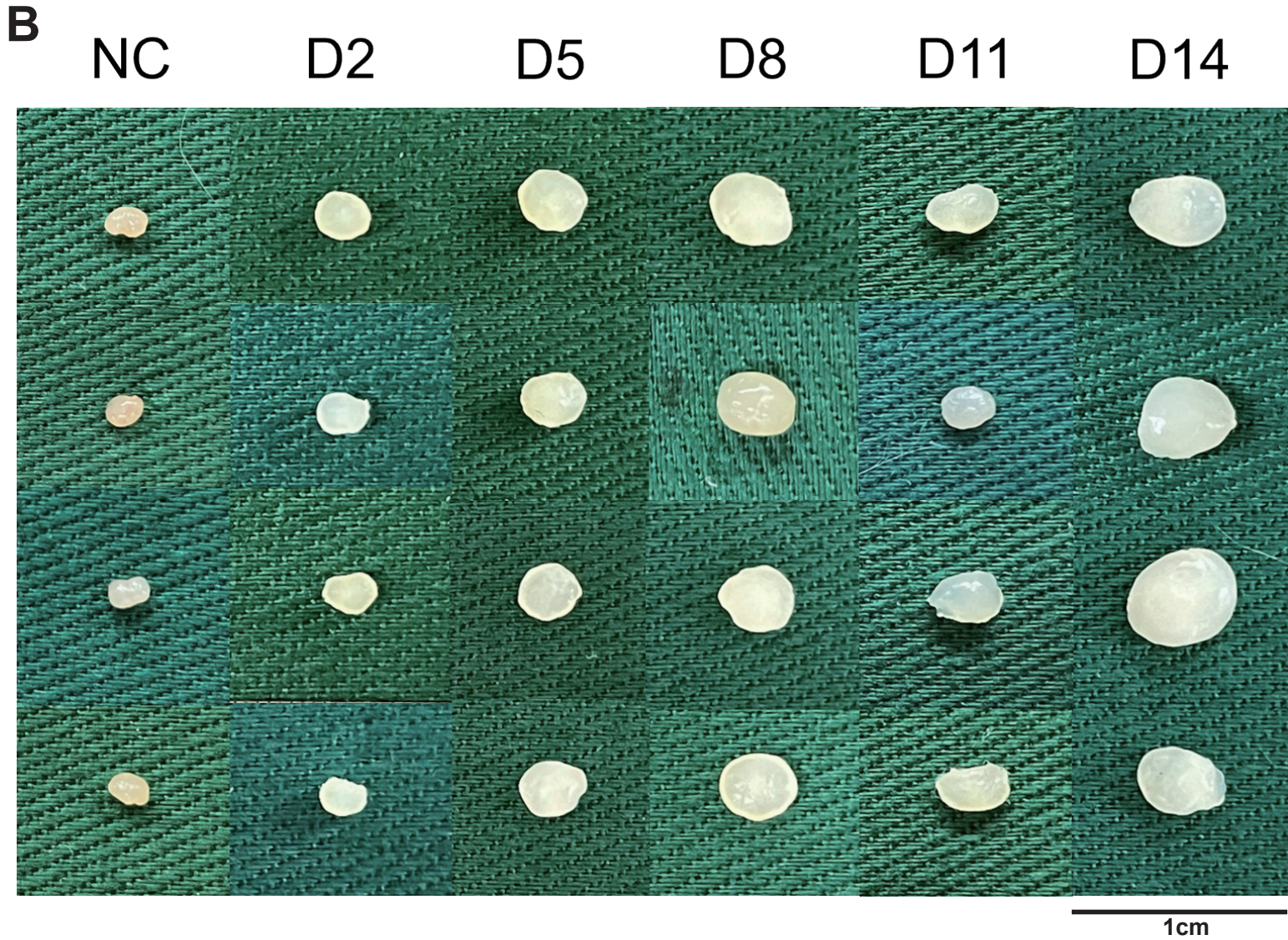

Supplement: Supplementary 1 — Figs. S1 to S11 [file research.0346.f1.zip › FigureS1.pdf]

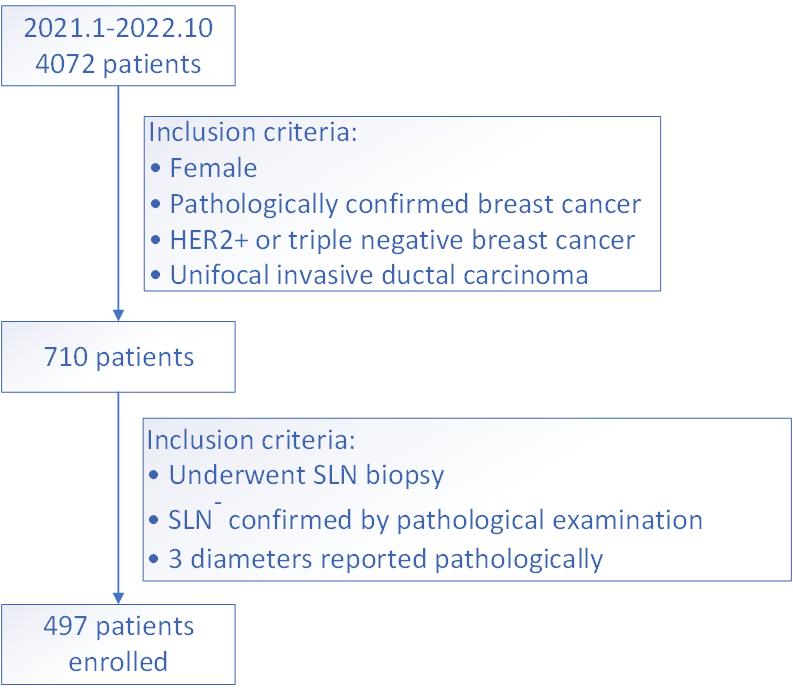

Supplement: Supplementary 1 — Figs. S1 to S11 [file research.0346.f1.zip › FigureS10.jpg]

**A**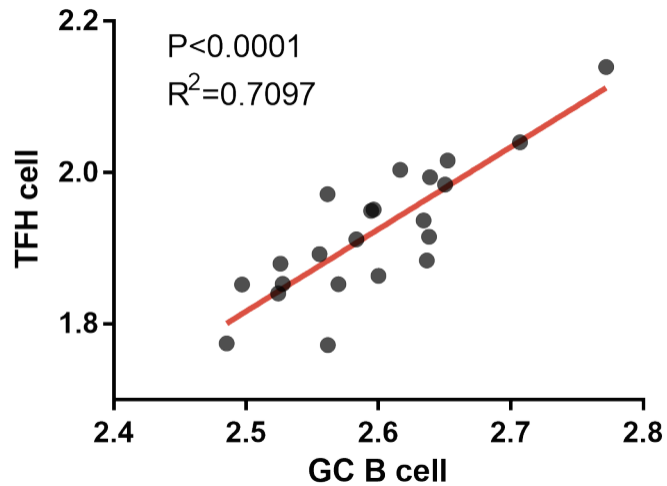**B**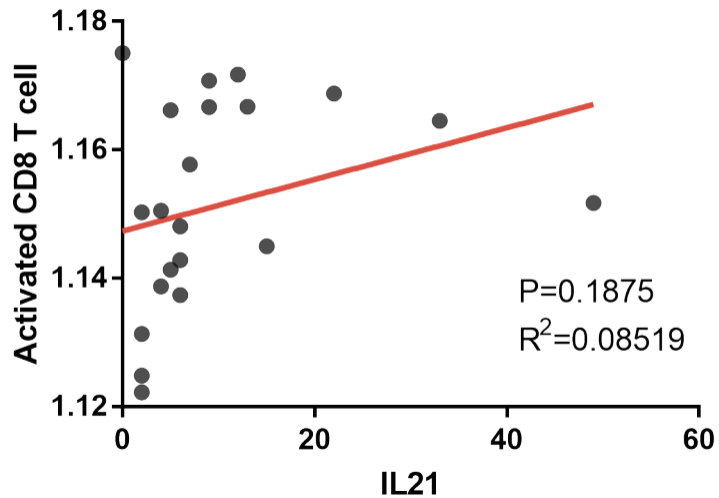

Supplement: Supplementary 1 — Figs. S1 to S11 [file research.0346.f1.zip › FigureS11.pdf]

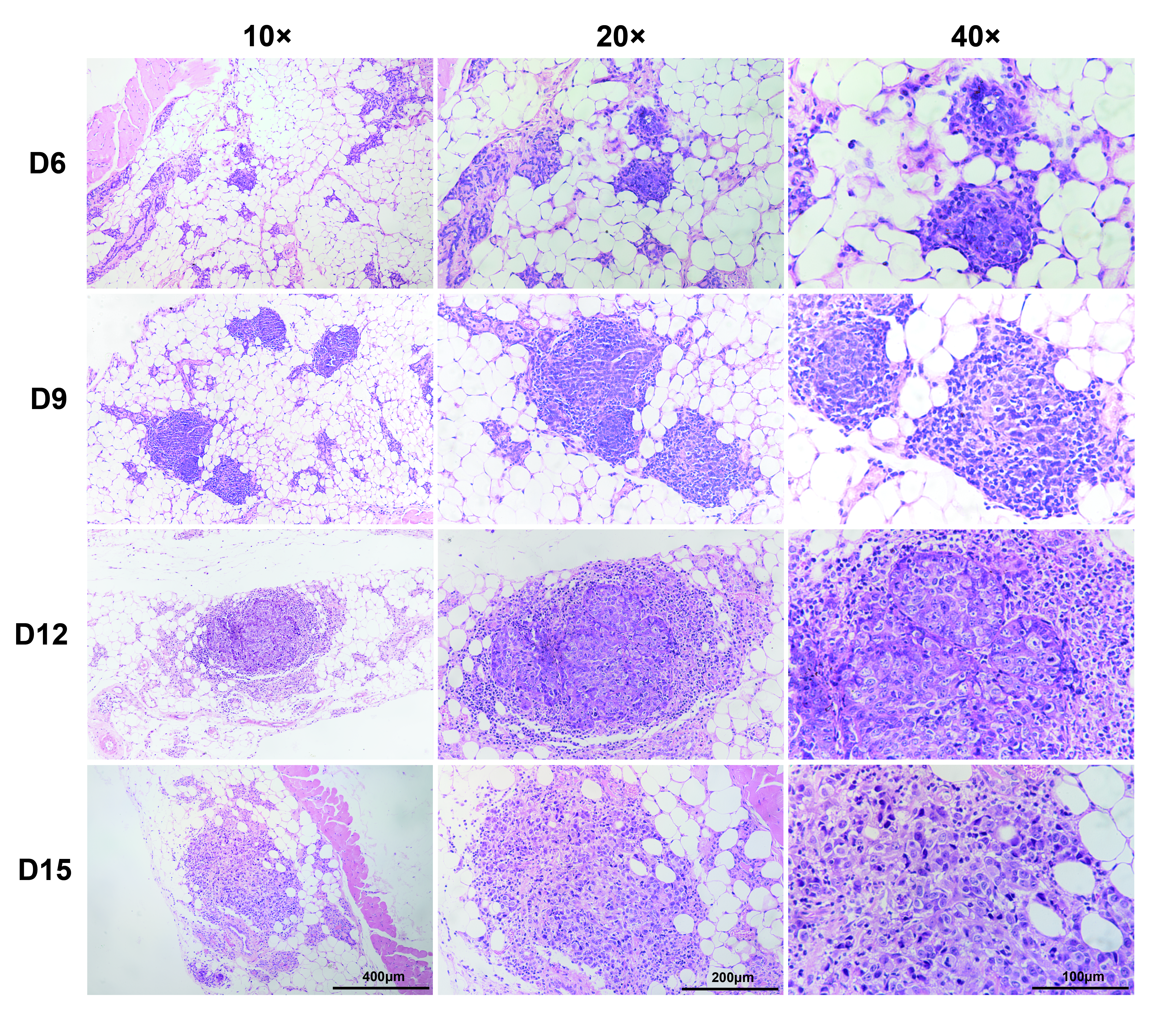

Supplement: Supplementary 1 — Figs. S1 to S11 [file research.0346.f1.zip › FigureS2.jpg]

A

KEGG

Reactome

D8  
vs  
NC

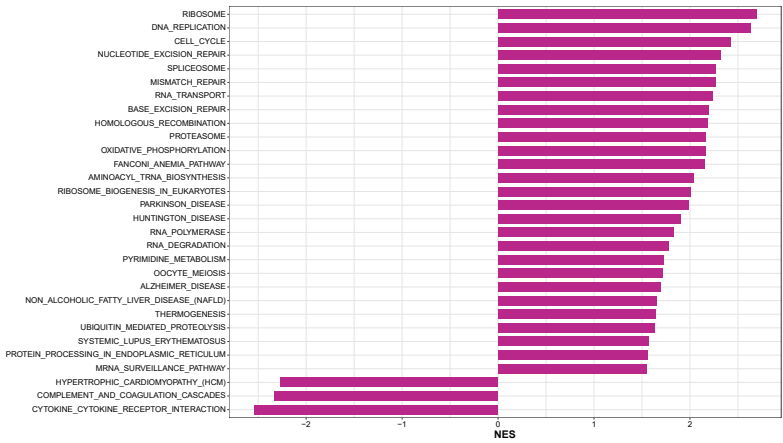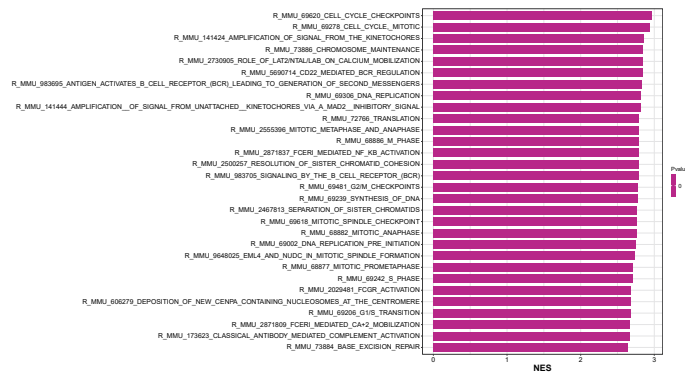

B

D11  
vs  
D8

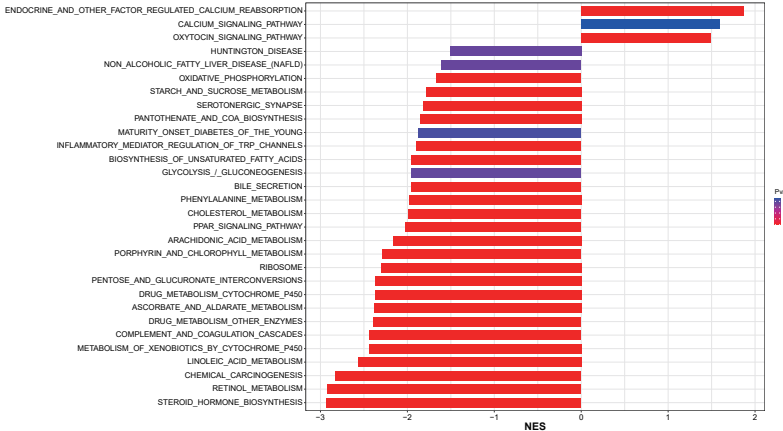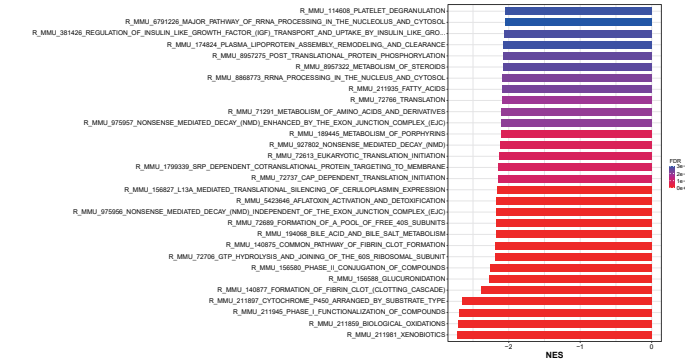

C

D11  
vs  
NC

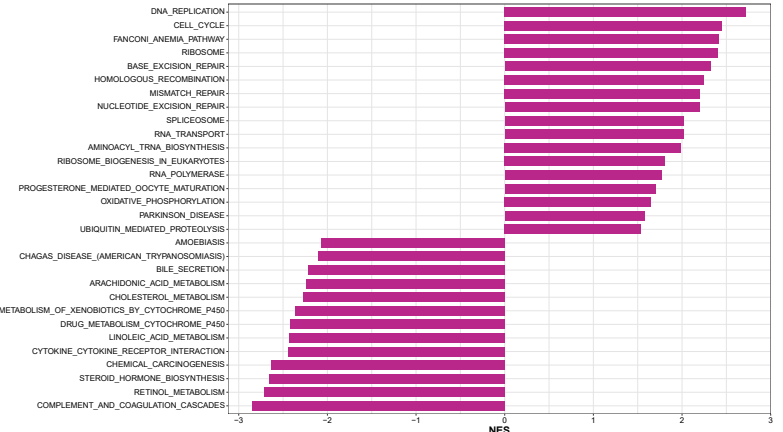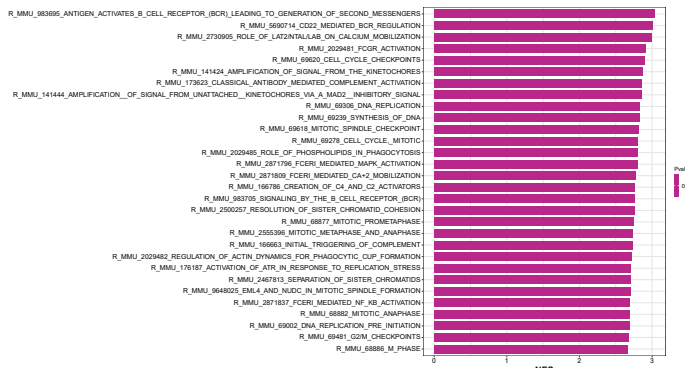

Supplement: Supplementary 1 — Figs. S1 to S11 [file research.0346.f1.zip › FigureS3.pdf]

10W

D8

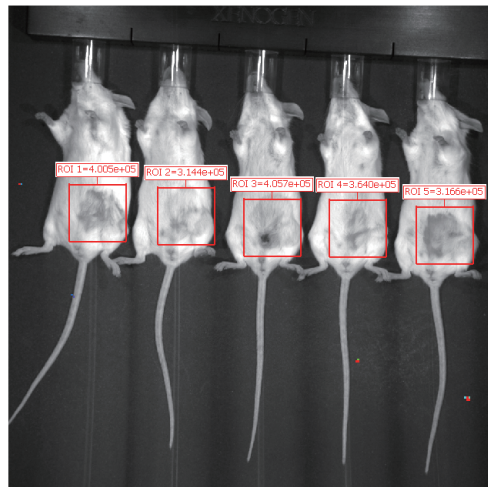

30W

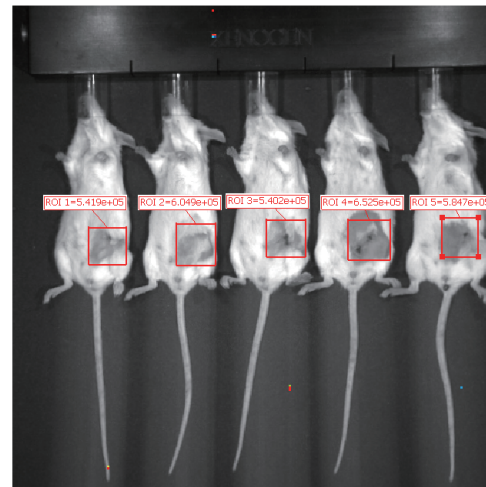

D11

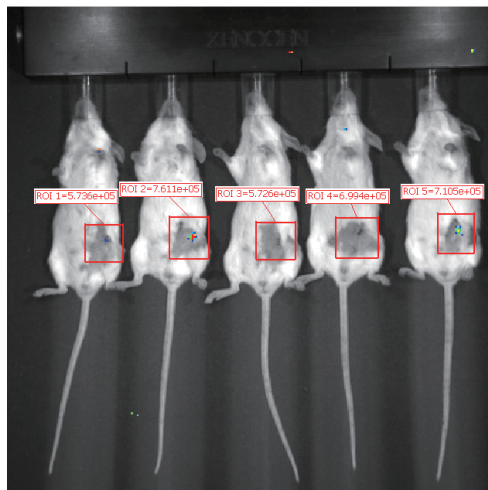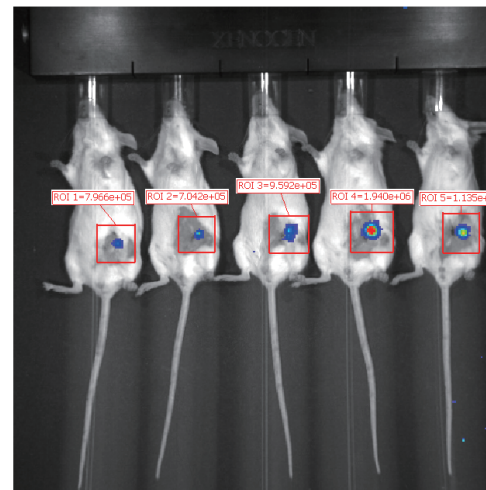

Supplement: Supplementary 1 — Figs. S1 to S11 [file research.0346.f1.zip › FigureS4.pdf]

**A**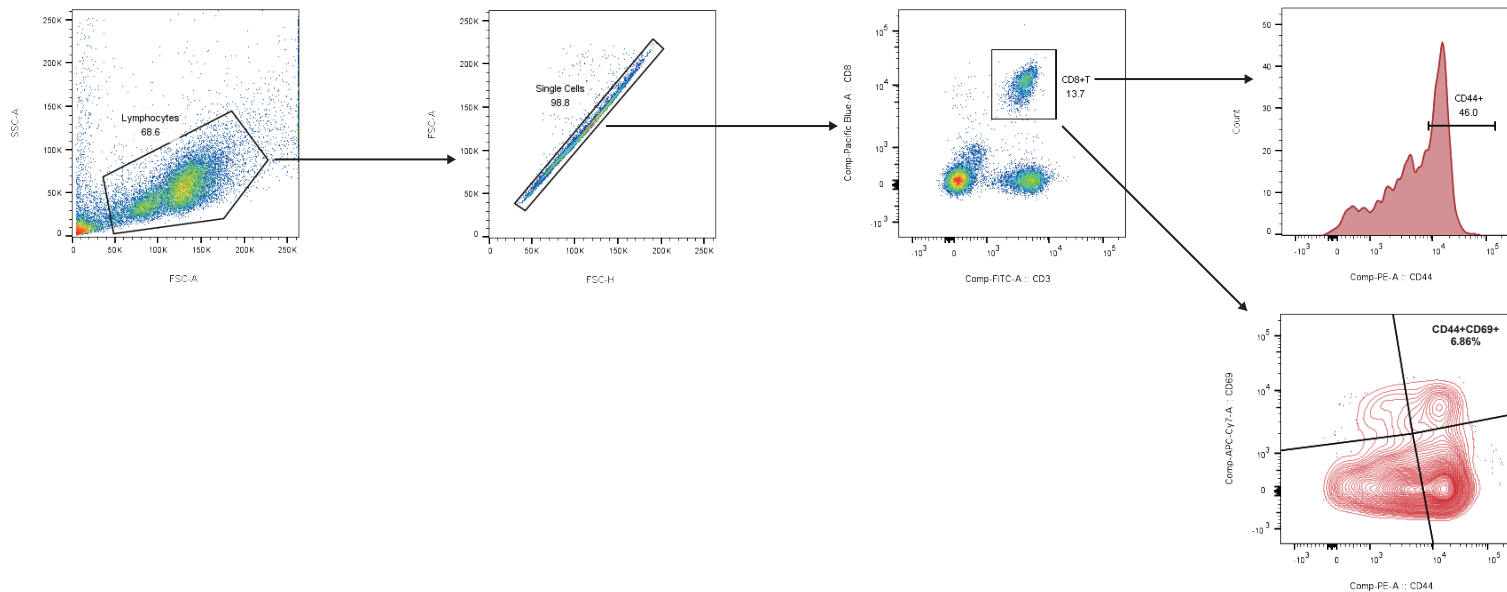**B**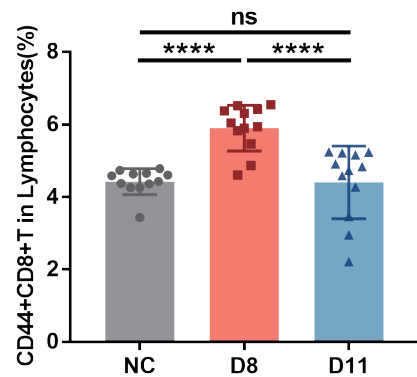**C**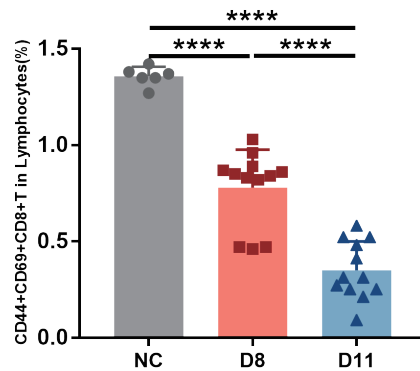

Supplement: Supplementary 1 — Figs. S1 to S11 [file research.0346.f1.zip › FigureS5.pdf]

A

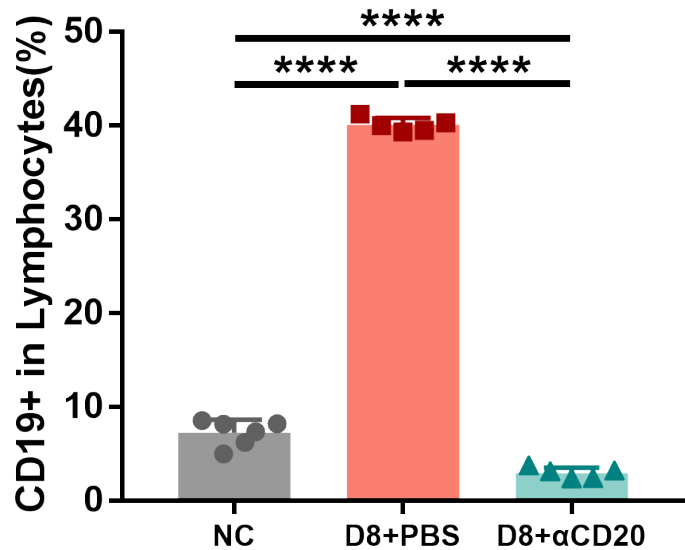

B

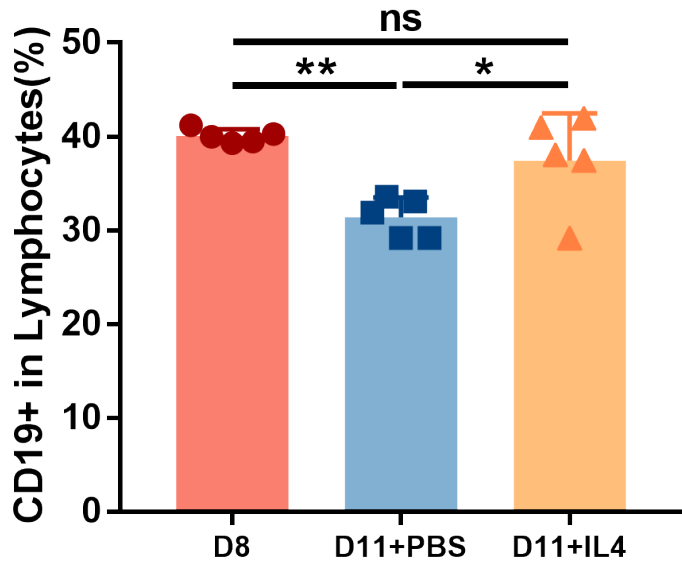

Supplement: Supplementary 1 — Figs. S1 to S11 [file research.0346.f1.zip › FigureS6.pdf]

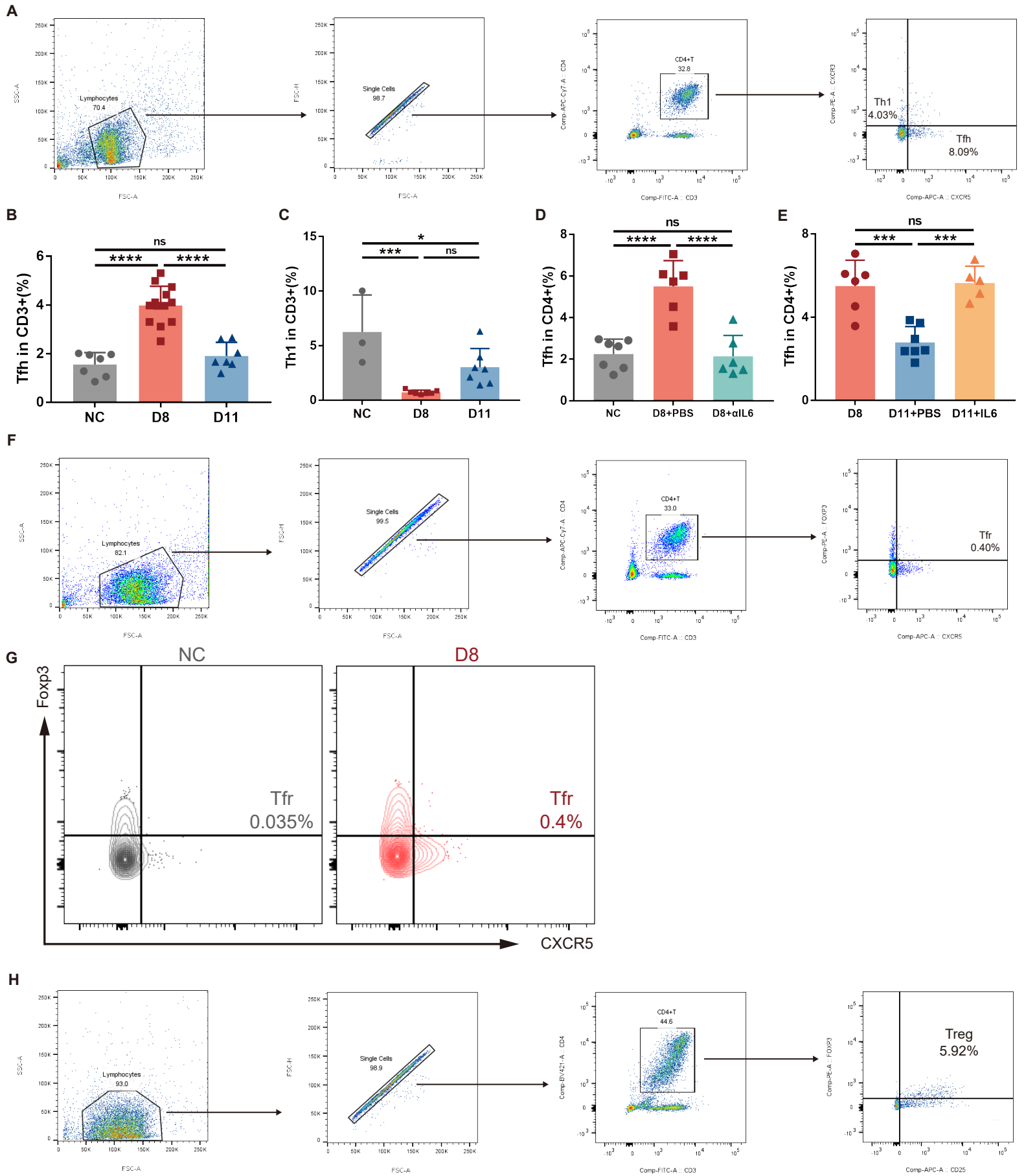

Supplement: Supplementary 1 — Figs. S1 to S11 [file research.0346.f1.zip › FigureS7.pdf]

**A**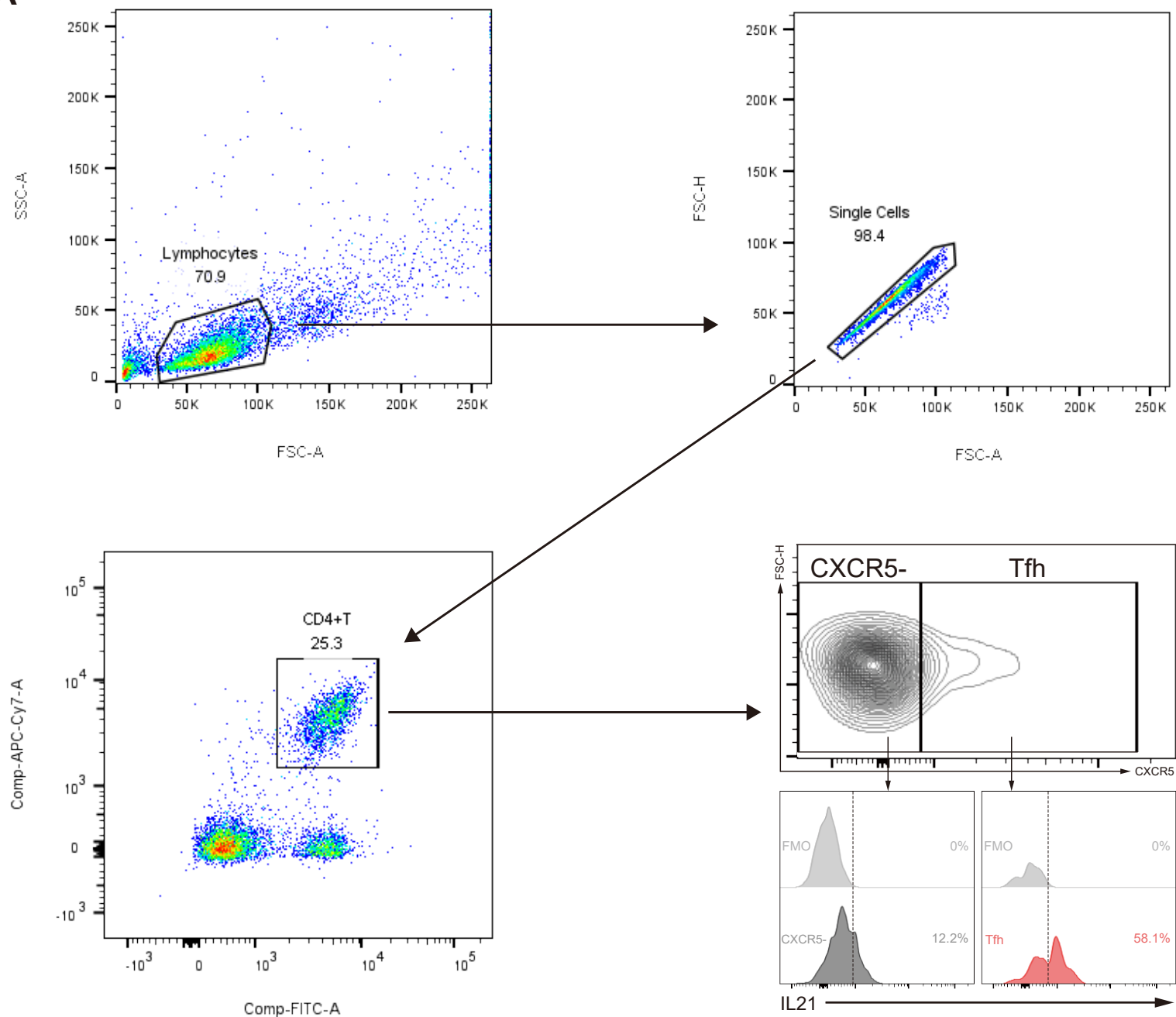**B**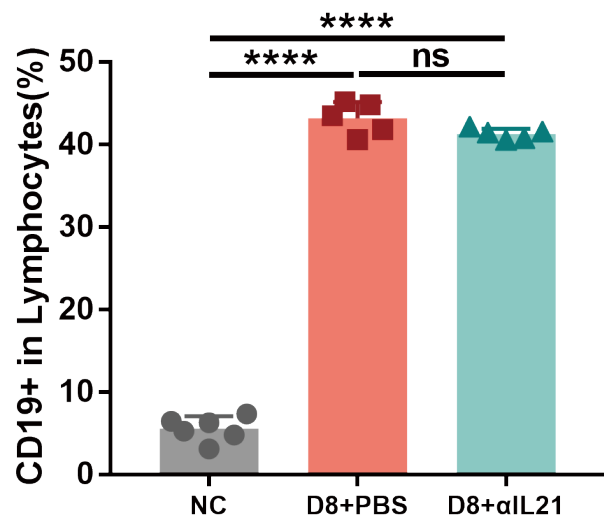**C**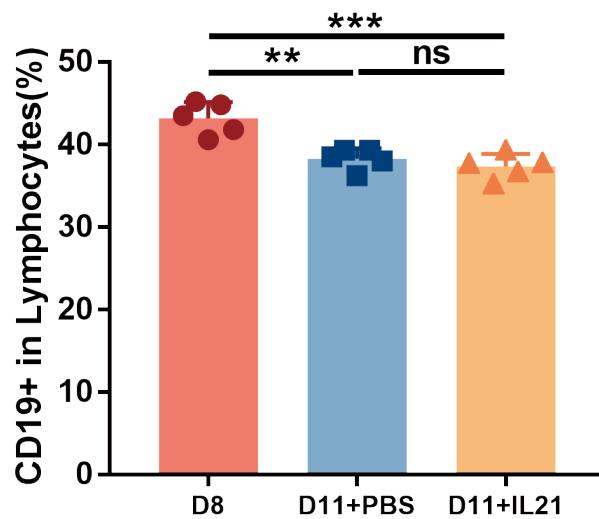

Supplement: Supplementary 1 — Figs. S1 to S11 [file research.0346.f1.zip › FigureS8.pdf]

**a**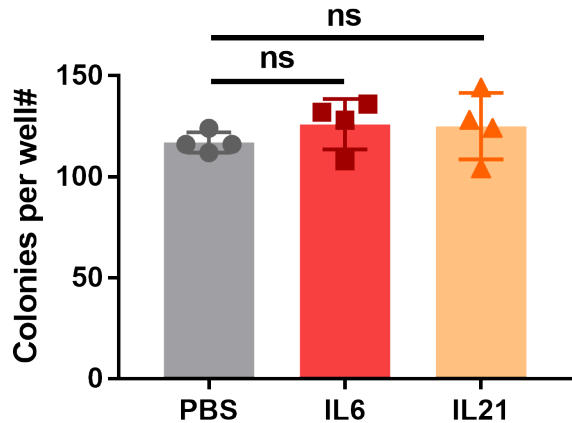**b**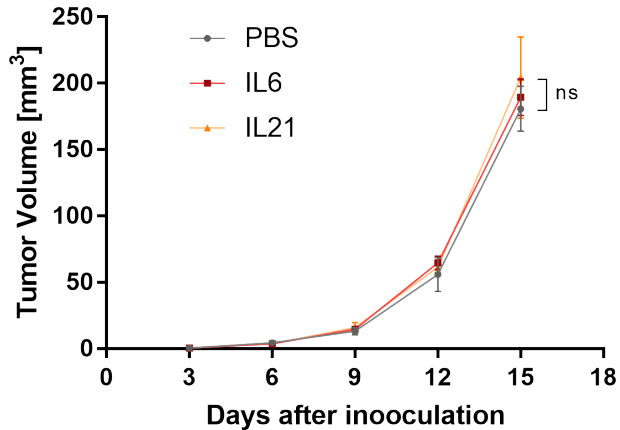

Supplement: Supplementary 1 — Figs. S1 to S11 [file research.0346.f1.zip › FigureS9 20240224.pdf]
